# Robust techno-economic optimization of energy hubs under uncertainty using active learning with artificial neural networks

**DOI:** 10.1038/s41598-025-12358-z

**Published:** 2025-07-26

**Authors:** Aya M. A. Heikal, Shady H. E. Abdel Aleem, Ragab A. El-Sehiemy, Almoataz Y. Abdelaziz

**Affiliations:** 1 Electrical Power & Machines Department, Faculty of Engineering, Ain Shams University, Cairo, 11517 Egypt; 2 Department of Electrical Engineering, Faculty of Engineering, Science Valley Academy, El-Obour City, Egypt; 3 Department of Electrical Engineering, Institute of Aviation Engineering and Technology, Giza, 12658 Egypt; 4 Department of Electrical Engineering, Faculty of Engineering, Kafrelsheikh University, Kafr El Sheikh, 33516 Egypt; 5 Sustainability Competence Centre, Széchenyi István University, Egyetem square 1, Győr, H-9026 Hungary; 6 Faculty of Engineering & Technology, Future University in Egypt, Cairo, 11835 Egypt

**Keywords:** Active learning approach, Artificial neural networks, Energy hubs, Muti-objective optimization framework, Sustainability, Techno-economic trade-off, Uncertainty, Energy science and technology, Engineering

## Abstract

Energy hubs (EHs) are considered a promising solution for multi-energy resources, providing advanced system efficiency and resilience. However, their operation is often challenged by the need for techno-economic trade-offs and the uncertainties related to supply and demand. This research presents a multi-objective optimizing framework for EH operations tackling these techno-economic aspects under uncertainty. Utilizing artificial neural networks (ANN)-based active learning (AL), the proposed approach dynamically enhances the model’s capability to achieve optimal scheduling and planning while considering complex, fluctuating energy demands and system constraints. The optimization approach under uncertainty provides robust predictive abilities across various scenarios, allowing the system to optimize energy management effectively, enhancing operational efficiency while minimizing overall energy losses, costs, and emissions. Results demonstrate significant improvements in system reliability, cost efficiency, and flexible operation, validating the effectiveness of ANN-based AL to optimize EHs management and ensure sustainable operation complexities. The AL algorithm enhances the ANN model’s predictive ability, resulting in a 57.9% decrease in operating costs and a 0.010682 loss of energy supply probability (LESP) value. It ensures energy efficiency while sustaining system flexibility, adapting to frequent load dynamics and intermittent renewable energy supply. The algorithm minimizes electrical and thermal deviations, achieving a balance of flexible operation with efficient energy management. Despite uncertainties and intermittent renewable energy supply, the AL optimizes renewables utilization and demand adjustments, reducing energy losses, costs, and emissions by 80.3The optimized system achieves an output of 13,687.8 kW per day. The system’s implementation is performed using MATLAB R2023b software, ensuring precision and efficiency.

## Introduction

### Background and motivation

**Fig. 1:**
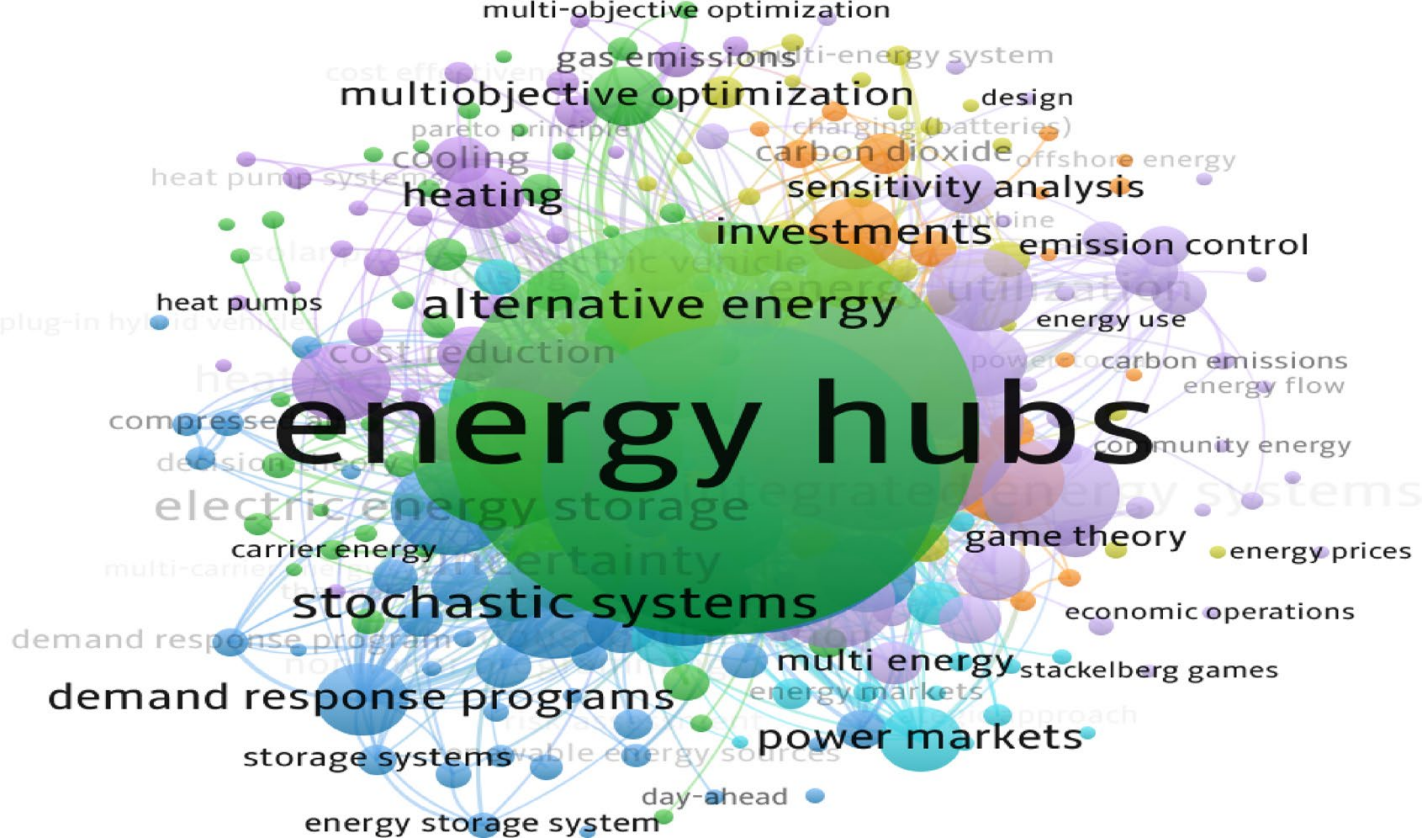
A schematic overview of EH’s distribution chart and optimization approaches.

Renewable energy sources (RESs) utilization has become an inevitable trend of the global energy transition due to resource reserve constraints and the environmental issues associated with fossil fuels^1^. As a green energy future with wide prospects, energy hubs (EH) are becoming a promising target for energy transition and have been improved rapidly. The fuel price policy and energy price mechanisms have also encouraged the rapid growth of EH^2^. The share of RESs in the energy sector is expected to increase from 30% in 2023 to 35% in 2025. In addition, global energy consumption is predicted to rise by 4% in 2024 and 2025, which represents the highest rise in decades. Notwithstanding the increasing utilization of RESs, the overall rise in demand still exceeds the share of RESs in the energy sector, resulting in issues with environmental concerns toward $\:{\text{C}\text{O}}_{2}$ emissions and fossil fuels power generation. Mainly, it is recorded that the greenhouse gases (GHG) have risen by 0.72 kt $\:{\text{C}\text{O}}_{2}$/year in 2024 compared to 2020 and 2023, which recorded emissions at about 0.58 kt $\:{\text{C}\text{O}}_{2}$/year. Developed technologies for smart grids have provided new chances for integrating multiple energy resources into power networks^3^. The RES nature and environmental conditions variations cause intermittency and system output fluctuations, which could hinder the grid from meeting consumer needs^1^. The stability of the power system depends on the accurate estimation of electrical demands. Therefore, developing an accurate forecast model for EHs power generation is a helpful way to address this issue^6^. Various approaches are performed in research studies to overcome these problems and enhance the performance of EH to increase its efficiency, as depicted in Fig. 1:

- Accurate load prediction ensures EH stable operation^7^.
- Integrated demand response (IDR) sets power consumption at peak hours to ensure EH’s optimal operation^8^.
- Multi-carrier EH optimal scheduling ensures economical and stable performance^9^.
- Muti-objective functions framework encourages optimal operation for EH^10^.
- Demand response with uncertainties approaches can help ensure accurate load forecasting, ensuring power system reliability^11^.

**Fig. 2:**
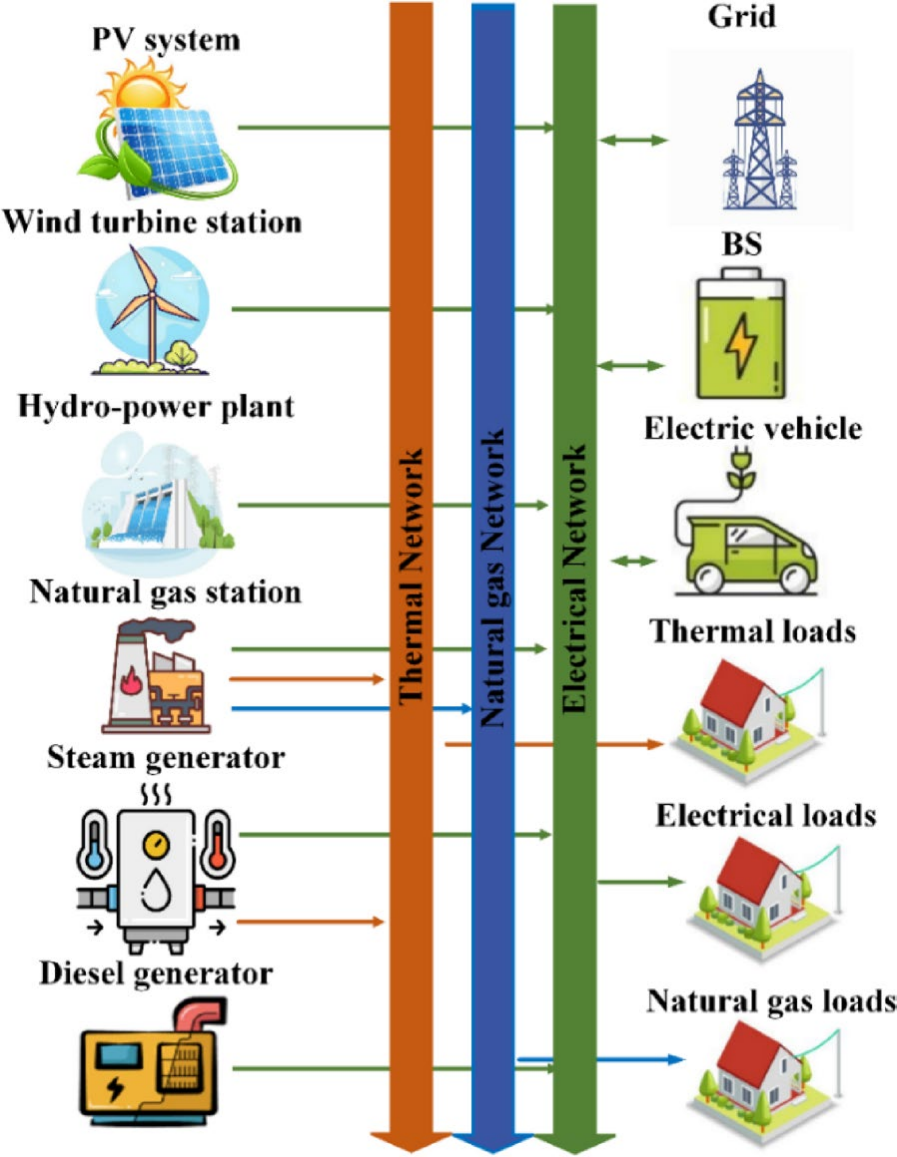
The proposed EH structure.

The EH aims to improve the performance of power systems without environmental and economic issues, making it a vital participant in the energy sectors and facilitating integration between advanced power management issues and energy storage solutions^4^. EH has paid attention to its environmental aspects and power sustainability. It integrates the electricity grid, RESs, and gas network, reducing pollution and ensuring system reliability^4^. Optimal scheduling and planning for EH are vital for system structures, as they ensure power sustainability and minimize overall operating costs. Various models have been proposed to perform uncertainty scenarios, including the particle swarm optimization (PSO) approach, benders decomposition algorithm, probabilistic, stochastic optimization, and robust methods with the presence of Photovoltaic (PV) modules and wind turbines (WT) in power systems^5^. In a restructured energy market, decisions made by consumers, operators, and generators may be impacted by integrating several units, such as combined heat and power (CHP), battery storage units (BS), steam generators (SGs), and power electronic devices^6^. Optimization algorithms may increase network performance, resilience, and flexibility through demand response plans and energy storage systems, helping EH achieve low operating costs and minimum environmental concerns^7^. Improving the predictive capabilities of the power system is imperative for optimizing the efficient functioning of the EH systems. Various factors, such as weather conditions and the unpredictable nature of RESs, can adversely affect EHs output^6^. Effectively managing these factors poses a significant challenge in maintaining system reliability^8^ and enhancing its capacity to meet electricity demands^9^. EH systems are integrated with a diverse range of components to enhance EH performance, which includes reducing energy losses, ensuring operational efficiency, and minimizing emissions and costs^10^. These components include BS, RESs, hydropower plants, diesel generators (DGs), steam generators, and natural gas stations (NG) connected to the grid alongside electric vehicles (EVs), as depicted in Fig. 2.

Sustainability is of chief significance^8^ especially concerning EH systems and the holistic assessment of their environmental, social, and economic ramifications to boost energy efficiency, curtail emissions, and cut costs. Although incorporating various RESs introduces complexities, BS techniques and demand response (DR) strategies bolster system adaptability^8^. This integration aligns sustainability indicators with the challenges of techno-economic trade-offs in EHs, facilitating a thorough examination of how EHs systems streamline energy usage, diminish emissions, and achieve cost efficiencies, notwithstanding the hurdles posed by multiple RESs. Optimizing EH operations according to demand response and BS units can increase reliability while decreasing costs and pollution. However, The concerns involve evaluating risk, considering rising electrical needs, and recognizing the optimal operation approach for EHs^6^.

### Literature review

Several researchers have improved techniques that enhance EHs operation to achieve optimal scheduling and increased demand prediction ability for consumers’ requirements, as presented in Table 1. Recent EH advancements have focused on improving optimal scheduling, demand prediction, and sustainable power management through various modeling and optimization approaches. The increasing penetration of RESs, EVs, and demand-side flexibility has significantly impacted EH designs, highlighting the need for efficient and robust planning under uncertainty. The integration between RESs, such as solar PV, wind turbines, and hydro-power plants, plays a critical role in enhancing the economic and environmental performance of EHs^11^. These technologies, combined with energy storage systems, enable demand-side flexibility and contribute to system reliability^12^ which emphasizes the role of RESs in reducing procurement costs and enhancing system reliability through strategic energy consumption. The authors in^13^ improved their energy hub scheduling framework by integrating electrical and thermal storage system management and demand response strategies and proposed an innovative energy conversion pathway for renewable energy diversification.

Several studies have evaluated the coordinated operation of these components using model predictive control and multi-objective optimization. Meanwhile^14^, explored control strategies for storage systems in hub substations under site constraints and renewable variability. Artificial neural networks (ANNs) have gained prominence for load prediction, energy demand forecasting, and real-time control due to their ability to model nonlinear and dynamic behavior in complex systems. Deep learning methods, such as deep neural networks (DNNs) and convolutional neural networks (CNNs), have shown promise in improving short-term load forecasting accuracy^15^. ANN-based approaches are widely used for load prediction, although their performance depends heavily on hyperparameter tuning^16^. Recent hybrid approaches, including CNNs^17^ PSO-enhanced ANN^18^ and deep learning frameworks^19^ have significantly improved forecasting accuracy and computational performance.

Emerging works also emphasize the necessary need to balance capital and operational expenditures with environmental sustainability^20^. Life cycle analysis, component degradation models, and predictive maintenance strategies have been embedded in EH designs to improve long-term viability^21^. Several studies have evaluated the coordinated operation of these components using model predictive control and multi-objective optimization. The authors in^22^ improved two-layer hosting capacity models for EV and RESs integration, resulting in cost savings of up to 12.3%. Hierarchical EH planning approach for optimizing MES configurations, using multi-objective optimization and Pareto front technique, improves cost-effectiveness and reliability^23^.

EHs offer a promising framework for the optimal coordination of multi-energy systems (MESs), enabling efficient management of integrated energy carriers. Although MESs pose challenges such as high capital costs and operational uncertainties including equipment failures and fluctuating demand, they are gaining traction due to their potential for enhancing energy efficiency and reducing carbon emissions^24^. However, further research is needed to address uncertainties and ensure reliable, adaptive energy management strategies in evolving energy power sectors. Robust and stochastic optimization frameworks have been widely adopted to address uncertainties in energy demand, generation, and market prices. Techniques such as information gap decision theory (IGDT)^1^ two-stage stochastic programming^25^ and scenario-based planning^26^ have been applied to reduce operational risk and ensure cost-effective scheduling.

Robust and stochastic optimization frameworks have been widely adopted to address uncertainties in energy demand, generation, and market prices. Techniques such as information gap decision theory (IGDT)^1^ two-stage stochastic programming^25^ and scenario-based planning^26^ have been applied to reduce operational risk and ensure cost-effective scheduling.

Distributed energy resources (DER), combining technologies like CHP, WT, PV, and power-to-gas (P2G) systems have been modeled using tools such as DER-CAM and HOMER^27^ but many studies highlight the need for more advanced models that account for uncertainty and component interaction in dynamic environments. have been used to explore optimal design and economic efficiency; an integrated approach that combines renewable and non-renewable resources through EH modeling remains largely unexplored. According to research, IDR software can achieve load demand profile optimization and increase system reliability. Several works focus on managing uncertainty and risk in EH operations. The authors in^25^ introduced a two-level optimization framework that reduced total EH cost using demand response and integrated energy programs. The integration of CHP units, EVs, BS, and P2G systems^9^ has been explored to increase energy efficiency and reduce reliance on the grid^28^. In addition, risk-aware and stochastic models have also been used to enhance resilience, considering uncertainties in RES availability, energy prices, and equipment failures^9^.

The authors in^29^ proposed a two-stage stochastic programming framework that was developed to optimize the participation of a virtual EH in day-ahead electricity markets while minimizing real-time imbalance costs. The proposed approach accounts for uncertainties and enhances decision-making under variability. Furthermore, the analysis confirms that adopting a risk-averse optimization strategy may lead to reduced profits but significantly improves the system’s resilience and preparedness against market and operational fluctuations. The authors in^30^ presented a robust and sustainable day-ahead scheduling model that was proposed for a zero-energy hub by integrating stochastic programming with information gap decision theory (IGDT). This hybrid approach effectively manages uncertainty while ensuring operational reliability. The results demonstrate that incorporating just 10% demand-side flexibility leads to a 15.6% reduction in operational costs. Additionally, the risk-based modeling framework highlights the sensitivity of costs to different levels of uncertainty, offering valuable insights for resilient energy management. The authors^31^ in A risk-aware two-stage stochastic optimization model was proposed for managing hybrid multi-microgrid systems under uncertainty. The model effectively captures the impact of variable conditions on system performance. Results show that while the risk-averse strategy leads to an 8.4% reduction in overall profit, it successfully eliminates downside financial risk, thereby enhancing the financial security and reliability of system operations.

The authors in^32^ developed a robust planning framework for an energy hub (EH) incorporating hydrogen fueling and battery swapping infrastructure, utilizing a scenario-based modeling approach combined with Conditional Value-at-Risk (CVaR) to effectively handle uncertainties. The results demonstrate that implementing load redistribution can regulate approximately 10% of fluctuations in the load profile, leading to a 4.55% increase in overall profit. This highlights the framework’s ability to enhance both economic performance and operational stability in uncertain environments. The authors in^33^ introduced a stochastic optimization framework for planning an advanced energy hub (EH) system integrated with flexible demand-side strategies. The model employs a scenario-based approach, including scenario reduction and downside risk assessment, to address both risk-neutral and risk-averse decision-making perspectives. The results indicate that the risk-averse strategy effectively reduces the expected downside risk by nearly 100%, while the implementation of a time-of-use pricing strategy achieves a 19.02% reduction in operational costs. These outcomes highlight the framework’s strong potential for mitigating the impacts of uncertainty and enhancing cost-effective energy management. Overall, ANNs have proven effective across multiple power system applications—including wind speed and PV output forecasting by enhancing control, prediction accuracy, and operational efficiency. ANN have proven effective in forecasting and optimizing EH operations under uncertainty. By training on historical data that captures variations in weather, energy prices, demand fluctuations, and outages, ANNs demonstrate strong adaptability across diverse demand response strategies. Studies show that ANNs outperform other intelligent optimization methods in predicting EH output power and maintaining a reliable balance between supply and electrical/thermal demands. Specifically, ANNs excel in converting meteorological data such as solar irradiance, temperature, wind speed into accurate power generation forecasts for PV and wind turbine systems. Their iterative training and ability to handle nonlinear, complex systems make them well-suited for dynamic EH environments. Additionally, accurate forecasting is critical for power planning, especially considering uncertainties in renewable output, market volatility, and demand surges. As EH systems integrate multiple renewable sources and face increasing operational complexities, ANN-based models offer a promising solution for enhancing economic performance, energy security, and system reliability.

### Contributions and novelty

This study evaluates the proposed EH optimization framework across three scenarios: (i) a single-objective optimization approach while accounting for operational uncertainties, (ii) a multi-objective optimization framework incorporating uncertainty into the decision-making process, and (iii) implementing the ANN-based active learning (AL) approach within the multi-objective optimization framework under uncertainty, aiming to enhance adaptability and predictive performance. The system integrates diverse energy sources such as PV systems, WT, hydropower, NG, DGs, SGs, and CHP units, alongside grid-connected EVs and BS units. The findings highlight the framework’s adaptability, efficiency, and resilience in optimizing EH operations under varying uncertainty conditions. This work aims to:

- Tackles multi-objective challenges by addressing the trade-offs between cost efficiency, system reliability, and resilience while enhancing overall performance and operational efficiency.
- optimizes the integration of RESs, reduces supply-demand imbalances, and mitigates peak load issues through robust uncertainty management, resulting in a highly adaptive and efficient energy management system.
- Analyzes the impact of uncertainties on the performance of EHs using ANN with advanced forecasting capabilities to predict energy demand based on dynamic factors such as weather variations, energy price fluctuations, unplanned outages, and changing load patterns. The incorporation of gas distribution factors (GDFs) into the analysis provides deeper insights into the interactions between gas generation units and overall system operations, demonstrating superior accuracy and robustness in predictive modeling.
- Integrates AL with ANN to adapt under uncertain operating conditions, allowing the model to learn from dynamic system behaviors continuously. This integration enhances the predictive accuracy of the ANN by leveraging high-quality, relevant data, improving its responsiveness to sudden changes and uncertainties. Consequently, the proposed framework enables a more resilient and adaptive energy hub capable of addressing diverse operational challenges.
- Additionally, this study performs a comprehensive evaluation of innovative solutions to tackle the techno-economic challenges in power systems, offering a strategic framework to optimize operational efficiency, enhance system reliability, and achieve cost-effectiveness while addressing environmental and sustainability goals. It focuses on minimizing operating costs, carbon emissions, and energy losses while minimizing the loss of energy supply probability (LESP) and reducing deviations between predicted and actual energy demands, while enhancing daily profits, peak-hour demand management, and long-term scalability. It offers a sustainable solution to address increasing energy demands and environmental objectives, providing valuable insights for the future development and practical implementation of energy hubs.

### Organization

The remainder of this research is structured as follows: Sect. “Problem definition, EH configuration, and uncertainty approach” discusses the problem definition, configuration of EHs, and approach to modeling uncertainty. Section “EHs multi-objective optimization function” provides an in-depth explanation of the multi-objective optimization framework developed for the EHs. Section “Artificial neural network” introduces the artificial neural network methodology employed in this study. Section “Numerical results and their discussion” presents the numerical results, accompanied by a detailed discussion of their implications. Finally, Sect. “Conclusion” concludes the study by summarizing the key contributions, findings, and potential avenues for future research.

**Table 1:** Key contributions of the present work and comparative analysis with existing studies.

Ref.	Year	Main contributions
Mahmoudi et al.^34^	2025	• Presented an innovative multi-objective optimization framework for hybrid renewable energy systems based on gravitational search algorithm (GSA). • Achieved increase in renewable energy share by18.4% and 14.2% reduction in environmental damage.
Ahmed et al.^35^	2025	• Introduced a multi-objective optimization framework for hybrid renewable energy systems to minimize operational costs and reduce the loss of power supply probability.
Haider Muaelou et al.^31^	2024	• Presented a risk-aware two-stage stochastic optimization model for hybrid multi-microgrid system under uncertainties. • Validated that risk-averse strategy reduces profit by 8.4% but ensures zero downside financial risk.
Lubna et al.^33^	2024	• Presented a stochastic optimization framework for planning an advanced EH system integrated with flexible demand-side strategies • Used a scenario-based approach, scenario reduction, and downside risk assessment to capture both risk-neutral and risk-averse decision-making perspectives to mitigate uncertainty impacts. • Demonstrated a holistic solution for balancing sustainability, risk, and cost-efficiency
Zeng et al.^18^	2024	• Developed ANN-based model for predicting the daily solar panel energy output to improve solar energy utilization efficiency and validate it as a cost-effective tool for smart energy systems in diverse climatic regions.
Abdelfattah A. Eladl et al.^36^	2024	• Proposed a novel a robust multi-objective optimization framework for EH. • Achivied a 36.18% reduction in CO₂ emissions, a 14.22% increase in social welfare, and improved stability indicators, demonstrating its potential to guide sustainable, resilient, and high-performance energy hub development.
Haider et al.^30^	2023	• introduced a robust and sustainable day-ahead scheduling model for a zero-energy hub based on combining Stochastic Programming and information gap decision theory (IGDT). • Implemented 10% demand-side flexibility lowers operational costs by 15.6%, while risk-based modeling reveals cost sensitivity under varying uncertainty levels.
Norouzi et al.^29^	2023	• Developed an approach based on two-stage stochastic programming framework to optimize the participation of a virtual EH to optimize offers in day-ahead markets and minimize real-time imbalanced costs. • Validated that risk-averse optimization reduces profits but enhances the initiative against fluctuations.
Guodao Zhang et al.^37^	2023	• Developed an approach based on multi-objective optimization framework for microgrid energy hubs integrated RESs with DSM to enable flexible and reliable operation. • Empolyed multi-objective stochastic algorithm to solve the optimization problem and achieved up to 20.75% cost reduction and 7.94% emission reduction.
Davoudi et al.^38^	2023	• Proposed framework integrates multi-objective particle swarm Optimization (MOPSO) algorithm to minimize investment costs, operation costs, and enhance system reliability.
Sohrabi et al.^32^	2023	• Presented a robust planning framework for an EH with hydrogen fuelling and battery swapping infrastructure based on scenario-based modelling approach and Conditional Value-at-Risk (CVaR) to manage uncertainties. • Proved that load redistribution regulates around 10% of fluctuations in the load profile, resulting in a 4.55% increase in profit.
Maryam Mousavi et al.^39^	2022	• Developed a multi-objective scheduling model for an EH system based on a Genetic Algorithm. • Minimized operational costs and enhanced environmental performance of EHs, contributing to more sustainable and cost-effective energy system planning.
Mohammad et al.^40^	2022	• Developed a novel dynamic energy storage hub framework to enhance operational flexibility and system efficiency. • Reduced operational costs by 21.63% over a 3-day period.
Mansouri et al.^41^	2022	• Presented a multi-objective optimization model for EH system based on Mixed-Integer Non-Linear Programming (MINLP) to optimize its operation. • Reduced emission to 9.89% and enhanced system efficiency, leading to a 9.2% reduction in system losses.
Thang et al.^42^	2022	• Introduced a stochastic scheduling framework for multi-energy hub systems based on MINLP model to reduce operation costs to 2.0–14.5% and emission for 22.3%.
Mokaramian et al.^43^	2021	• Investigated the impact of Monte-Carlo and k-mines clustering approach to optimize EH performance. • Developed multi-objective function framework and achieved reduction around 4.8% in operating cost, 18.21% in carbon emission, and 10.10% in load deviation which in turn increase system reliability.
Chamandoust et al.^7^	2020	• Introduced a DSM approach integrated with the Monte Carlo technique for EH system in GAMS. • Developed an approach to minimize costs, reduce emission, LESP and load deviation as multi-objective function framework.
Current work	-	• Developed a new multi-objective function optimization approach to address the trade-offs between cost efficiency, system reliability, and resilience while enhancing overall system performance and its operational efficiency. • Introduced ANN forecasting algorithm with uncertainty. • Developed a hybrid algorithm combining features of ANN and AL approach. • Integrated GDF into the optimization enhances predictive accuracy and enables better coordination between gas generation and overall energy hub operations.

## Problem definition, EH configuration, and uncertainty approach

Due to rising energy needs and the need for sustainable solutions, EH effectively combines several energy resources to optimize dynamic operation on the generation side and with consumers.

### Problem definition

Currently, EH has challenges finding accurate predictions for electrical and thermal needs and regulating the suitable GDFs between NG, DGs, SGs, and CHP modules, especially with integrating RESs, including a grid-connected EV and BS, which increases these issues. Inexactitude in power management and insufficient gas distribution between energy resources can lead to inadequate required power to meet electrical and thermal needs, rising operating costs, increasing carbon emissions, especially with continuously changing energy prices, and the intermittent nature of RESs due to irregular weather conditions. Furthermore, the use of intelligent control strategies as model predictive control (MPC), which can enhance electrical and thermal needs predicting and improve gas distribution approaches due to not taking them into account, poses challenges for EHs to adapt to continuous variations in demand profiles and operation costs, which consequently pose difficulties in achieving their sustainable targets. Considering these challenges, this research aims to improve a comparative approach that integrates ANNs with an uncertainty approach to get accurate predictions for electrical and thermal needs while achieving suitable GDF. The target is to present a robust strategy that ensures efficient power management and decreases operation costs and carbon emissions, thus ensuring sustainability and reliability for EHs.

### EHs configuration

**Fig. 3:**
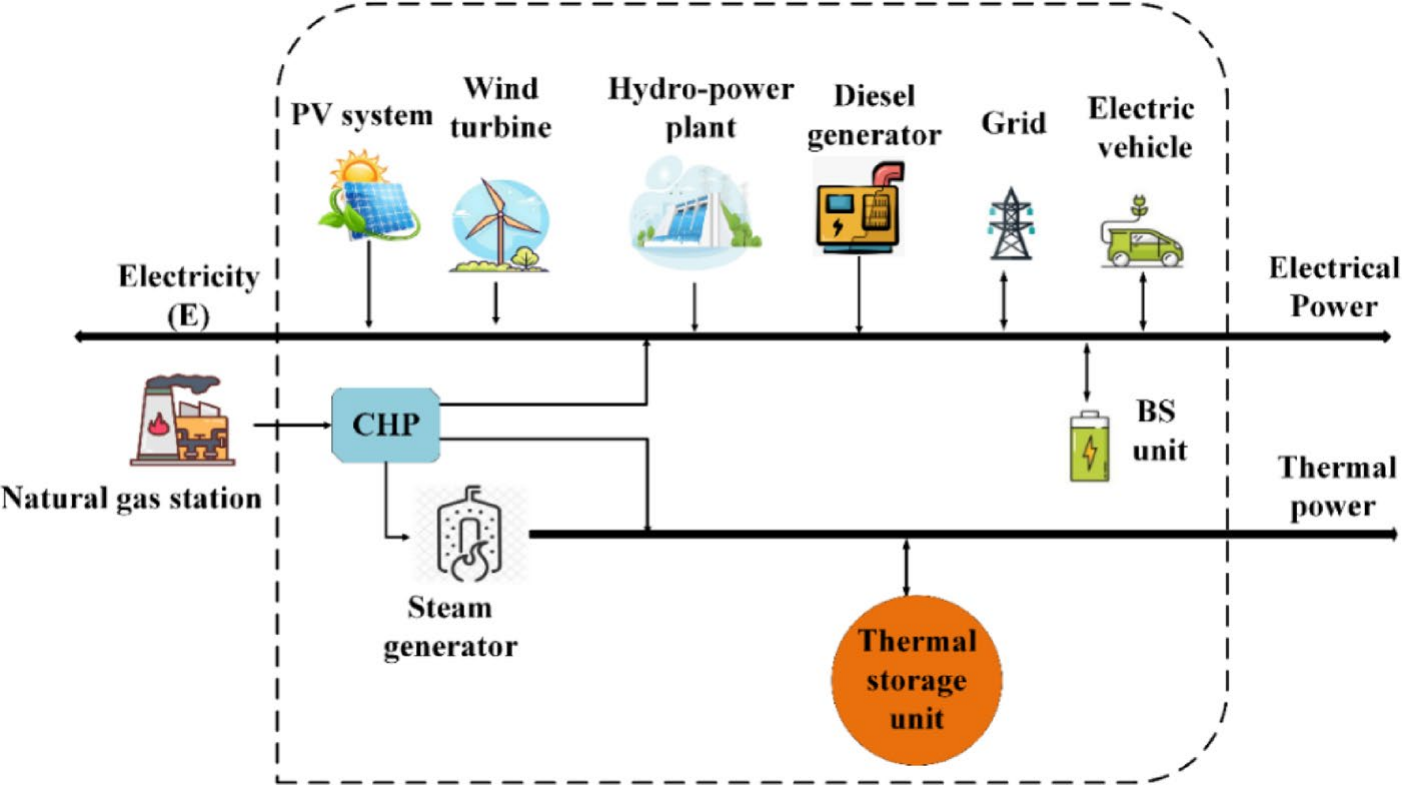
The detailed suggested EH configuration in this study.

The proposed EH has been set up using the approach described in this study. The EH under investigation is shown in Fig. 3. The EHs structure combines multiple inputs and outputs to meet thermal and electrical demands. It includes grid-connected EVs, renewable energy generation units, a CHP unit, NG, SG, and NG modules integrated with a hydroelectric power plant, and a BS unit. The NG station supplies the thermal network, while EVs act as a BS unit, saving surplus energy for low demands and compensating for peak loads, improving system performance and reliability.

The proposed method leverages input datasets and constraint parameters to refine the ANN-based AL framework. This intelligent adaptation strengthens the model’s predictive accuracy for future energy demands and enables more effective regulation of GDF across generating units. By continuously learning from system performance and incorporating updated training data, the ANN-AL approach delivers a resilient and adaptive solution for optimizing EH operations. The strategy aims to reduce operational costs, lower emissions, and minimize energy losses—ultimately enhancing efficiency, reliability, and the overall energy management of EH systems.

#### WT module

The WT module under uncertainty in wind speed using a stochastic approach^44^ is highly advanced in determining variation and continuous changes in weather conditions which impact output-generating power, are usually separated into four regions, as expressed in (1):

1 $$\:{P}_{wind\langle v\rangle}=\left\{\begin{array}{c}0\:\:\:\:\:\:\:\:\:\:\:\:\:\:\:\:\:\:\:\:\:\:\:\:\:\:\:\:\:\:\:\:\:\:\:\:\:\:\:\:\:\:\:\:\:\:\:\:\:\:\:\:\:\:\:\:\:\:\:\:\:\:\:\:\:\:\:\:\:\:\:\:\:\:\:\:\:\:\:\:\:\:\:\:\:\:\:\:\:\:\:\:\:\:\:\:\:\:\:\:\:\:\:\:\:\:\:v<{v}_{cut\_in}\\\:{P}_{r}\left(\frac{{\left(v\right)}^{3}-{\left({v}_{cut\_in}\right)}^{3}}{{\left({v}_{r}\right)}^{3}-{\left({v}_{cut\_in}\right)}^{3}}\right)\:\:\:\:\:\:\:\:\:\:\:\:\:\:\:\:\:\:\:\:\:\:\:\:\:\:\:\:\:\:\:\:\:\:\:\:\:\:\:\:\:\:\:\:\:\:\:\:\:\:\:\:{v}_{cut\_in}\le\:\:v<{v}_{r}\\\:{P}_{r}\:\:\:\:\:\:\:\:\:\:\:\:\:\:\:\:\:\:\:\:\:\:\:\:\:\:\:\:\:\:\:\:\:\:\:\:\:\:\:\:\:\:\:\:\:\:\:\:\:\:\:\:\:\:\:\:\:\:\:\:\:\:\:\:\:\:\:\:\:\:\:\:\:\:\:\:\:\:\:\:\:\:\:\:\:\:\:\:\:\:\:\:\:\:\:\:\:{v}_{r}\le\:v<{v}_{cut\_out}\\\:0\:\:\:\:\:\:\:\:\:\:\:\:\:\:\:\:\:\:\:\:\:\:\:\:\:\:\:\:\:\:\:\:\:\:\:\:\:\:\:\:\:\:\:\:\:\:\:\:\:\:\:\:\:\:\:\:\:\:\:\:\:\:\:\:\:\:\:\:\:\:\:\:\:\:\:\:\:\:\:\:\:\:\:\:\:\:\:\:\:\:\:\:\:\:\:\:\:\:\:\:\:\:\:\:\:v\ge\:{v}_{cut\_out}\\\:\:\:\:\:\:\:\:\:\:\:\:\:\:\:\:\:\:\:\:\:\:\:\:\:\:\:\:\:\:\:\:\:\:\:\:\:\:\:\:\:\:\:\:\:\end{array}\right.$$

where $\:\left(v\right)$, $\:{v}_{r}$,$\:\:{v}_{cut\_out}$, and $\:{v}_{cut\_in},$ represents the during-hour wind speed, rated wind turbine speed, cut-out wind turbine speed, and cut-in WT speed, respectively. $\:{P}_{r}\:\:$and $\:{P}_{wind\langle v\rangle}$ denote the rated and output wind turbine powers, respectively.

#### PV module

The PV module under uncertainty in temperature and irradiance using a stochastic approach^44^ is highly advanced in overcoming unpredictable factors such as variation in load demands and weather conditions. The PV module output power is represented by (2):

2 $$\:{P}_{PV}={N}_{PV}\:{P}_{R}^{PV}\left(\frac{GI}{{GI}_{0}}\right)\left(1-{T}_{coff}\left({T}_{ambient}-25\right)\right){\eta\:}_{v}{\eta\:}_{R}$$

where $\:{P}_{PV}$ and $\:{P}_{R}^{PV}$, represent the PV module output power and PV-rated power.$\:\:{\eta\:}_{v}$ and $\:{\eta\:}_{R},\:$represents the inverter and PV relative efficiency.$\:\:{N}_{PV}$, represents the number of PV stations. $\:{T}_{amb}$ and $\:{T}_{coff},\:$represents the ambient temperature and temperature coefficient. $\:{GI}_{0}$ and $\:GI$ are the standard and global solar irradiance under operating.

#### DGs module

DGs are reliable, flexible, and cost-effective alternatives for providing immediate electricity during emergencies or off-grid locations, making them popular in residential, commercial, and industrial settings. The fuel consumption^45^ $\:{F}_{DG}\left(t\right)$ required to obtain output power of the DGs is represented by (3), where $\:{P}_{n}^{DG}$, $\:{P}_{E}^{DG}$ are nominal power and output power, $\:{A}_{DG}$,$\:{B}_{DG}$ are intercept coefficient and fuel slope as follow:

3 $$\:\:\:{F}_{DG}\left(t\right)=\:{A}_{DG}\:{P}_{n}^{DG}+\:{B}_{DG}\:{P}_{E}^{DG}$$

#### NG, CHP and SGs modules

The NG module^43^ has electrical output power and thermal output power^7^ which is used through CHP. The electrical output power^7^ $\:{P}_{E}^{NG}$ and thermal output power $\:{Q}_{th}^{NG},\:$are represented by (4) and (5).

4 $$\:{P}_{E}^{NG}={\eta\:}_{E}^{NG}\:{F}_{NG}\left(t\right)$$

5 $$\:{Q}_{th}^{NG}={\eta\:}_{th}^{NG}\:{F}_{NG}\left(t\right)$$

where $\:{\eta\:}_{E}^{NG}and\:{\eta\:}_{th}^{NG}$ represent the NG electrical and thermal efficiency^46^ and $\:{F}_{NG}\left(t\right)$ represents NG gas input fuel.

The CHP unit provides electrical output power^47^ $\:{P}_{E}^{CHP}$ and thermal output power $\:{Q}_{th}^{CHP}$ as represented by (6) and (7), where $\:{F}_{CHP}\left(t\right)$ represents CHP gas input fuel, $\:{\eta\:}_{E}^{CHP}and\:{\eta\:}_{th}^{CHP}$ represent the SGs’ electrical and thermal efficiency.

6 $$\:{P}_{E}^{CHP}={\eta\:}_{E}^{CHP}\:{F}_{CHP}\left(t\right)$$

7 $$\:{Q}_{th}^{CHP}={\eta\:}_{th}^{CHP}\:{F}_{CHP}\left(t\right)$$

The SG module provides electrical output power^41^ $\:{P}_{E}^{SG}$ and thermal output power $\:{Q}_{th}^{SG}$ as represented by Eqs. (8) and (9), where $\:{F}_{SG}\left(t\right)$ represents SGs gas input fuel^48^ $\:{\eta\:}_{E}^{SG}and\:{\eta\:}_{th}^{SG}$ represents the SGs’ electrical and thermal efficiency.

8 $$\:{P}_{E}^{SG}={\eta\:}_{E}^{SG}\:{F}_{SG}\left(t\right)$$

9 $$\:{Q}_{th}^{SG}={\eta\:}_{th}^{SG}\:{F}_{SG}\left(t\right)$$

The gas input fuel is regulated, as expressed in Eqs. (10) and (11), by optimizing the GDF in EHs involving the NG module, DGs module, SG module, and CHP unit using an ANN with a stochastic approach to improve decision-making under operating conditions, considering efficiency, fuel costs, emissions, and power constraints.

10 $$\:x=\left[{x}_{NG}\:{x}_{DG}\:{x}_{SG}\:{x}_{CHP}\right]$$

where $\:x$ represents the GDF and its distribution between generating units, $\:{x}_{NG},\:{x}_{DG},\:{x}_{SG},\:{x}_{CHP}$ represents the share of gas distribution between the NG, DGs, SGs, and CHP modules.

11 $$\:x={x}_{NG}+\:{x}_{DG}+{x}_{SG}+\:{x}_{CHP}$$

The dataset serves as input to the ANN model, enabling optimal prediction of the GDF across generation units and demand profiles. By continuously training the model under varying operational conditions, the system can optimize EH performance, enhance reliability, and reduce costs and emissions. Effective ANN training involves minimizing validation and testing errors to ensure accurate forecasting under uncertainty. This method supports robust GDF optimization, promotes sustainable energy use, and improves integration of renewable sources and grid-connected EVs, all while achieving low operational costs and carbon emission.

#### Hydro-power plant

The hydro-power plant provides electrical output power^49^ $\:{P}_{E}^{Hydro}$ as represented by (12), where $\:{\eta\:}_{t}\:and\:{\eta\:}_{g}\:,\:$are the turbine and generator efficiency, $\:{\uprho\:}\:$ is the water density, $\:g$ is the gravitational acceleration, $\:Q$ is the water flow rate, and $\:H$ is the hydraulic height.

12 $$\:{P}_{E}^{Hydro}=\:{\eta}_{t}\:{\eta}_{g}\:{\uprho\:}\:Q\:g\:H$$

#### BS unit and grid-connected EVs

The integration of Lead Acid battery^50^ with grid-connected EVs in EHs aims to optimize power management, enhancing system stability and reliability^35^. The EV operates as a dynamic storage unit, which in turn participates in peak shaving to meet grid demands^50^. The BS ensures reliable storage energy utilization, decreasing dependency on fossil fuels. The energy exchange between the grid, EV, and BS is managed through an optimization approach to minimize costs and emissions. The state of charging (SoC) for BS unit $\:{SoC}_{BS}$ and for EVs $\:{SoC}_{EV}$^14^ denotes at time $\:\left(t\right)$, are represented as follows:

13 $$\:{SoC}_{EV}\left(t+1\right)={SoC}_{EV}\left(t\right)+\:{P}_{ch}^{EV}\:{\eta\:}_{ch}^{EV}\varDelta\:t-\:\frac{{P}_{dis}^{EV}\:}{{\eta\:}_{dis}^{EV}}\:\varDelta\:t$$

14 $$\:{SoC}_{BS}\left(t+1\right)={SoC}_{BS}\left(t\right)+\:{P}_{ch}^{BS}\:{\eta\:}_{ch}^{BS}\varDelta\:t-\:\frac{{P}_{dis}^{BS}\:}{{\eta\:}_{dis}^{BS}}\:\varDelta\:t$$

where $\:{P}_{ch}^{EV},\:{P}_{ch}^{BS},\:{P}_{dis}^{EV}$ and $\:{P}_{dis}^{BS}$ represents charging and discharging power flow, $\:{\eta\:}_{ch}^{EV},\:{\eta\:}_{ch}^{BS},{\eta\:}_{dis}^{EV}$ and $\:{\eta\:}_{dis}^{BS}$ represent charging and discharging efficiencies and $\:\varDelta\:t$ represents time interval.

### Uncertainty approach

The uncertainty approach involves performing accurate scenarios to explain uncertainties and challenges clearly. Accurate estimations of uncertainties should be based on realistic methodologies^51^ and each methodology requires an accurate design^1^. Uncertainty modeling simulates fluctuations in RESs, energy prices, and grid requirements^52^. Random distributions are used to adapt to the probabilistic nature of these variables. This study presents scenarios with defined probabilities to perform various system parameter uncertainties using ANN, uncertainty scenarios for energy needs, and gas distribution factors^16^. The ANN approach is trained on historical data sets to eliminate the mean squared error between predicted and actual outputs, enhancing its efficiency in reflecting uncertainties^53^. The ANN approach offers intelligent and adaptive solutions, continually learning from training data to improve electrical and thermal demand prediction, optimize GDFs, and maximize EH performance^18^. This aims for a more reliable and efficient operation that accommodates RES nature and continuous changes in energy prices while satisfying grid requirements^13^.

**Fig. 4:**
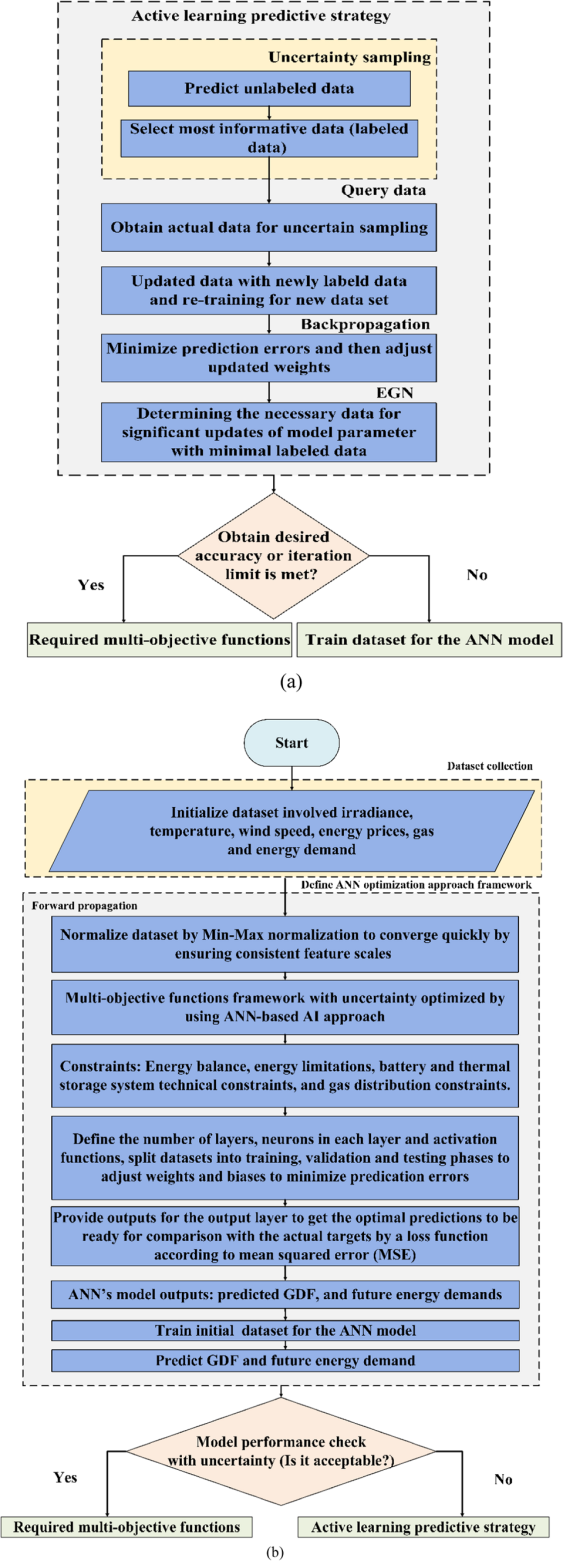
Model flowchart: **(a)** Active learning predictive strategy, and **(b)** ANN model framework.

ANN is a powerful tool for predicting intricate patterns in complex datasets, providing optimal control for power systems^54^. It helps address uncertainty and system complexity, enhancing EHs operation. The ANN-based AL approach^55^ uses historical data on uncertainty parameters, preprocessed as labeled datasets ($\:{D}_{l}$) and unlabeled datasets ($\:{D}_{u}$), and trained to minimize prediction errors using backpropagation. A stochastic approach generates multiple scenarios, generating a spectrum of potential outcomes. These scenarios are then optimized to minimize operational costs and emissions while considering environmental constraints. The integration of the AL approach with ANN, as depicted in Fig. 4, improves its predictive performance by selecting informative points in the dataset and continuously updating to learn from training data, and ensuring the EH’s accuracy with fewer labeled scenarios^56^ which in turn reduces complexity in computation and increases model efficiency^55^. The effectiveness of the AL approach lies in its ability to accurately identify and select the most informative data points, which in turn accelerates performance by iteratively adapting and updating $\:{D}_{l}$ and retaining new datasets^56^ to enhance system dynamic performance and achieve perfect predictions for GDF and future electrical and thermal demands. Due iterative method ensures the system operates efficiently under operating conditions while ensuring EH reliability while considering techno-economic aspects.

## EHs multi-objective optimization function

**Fig. 5:**
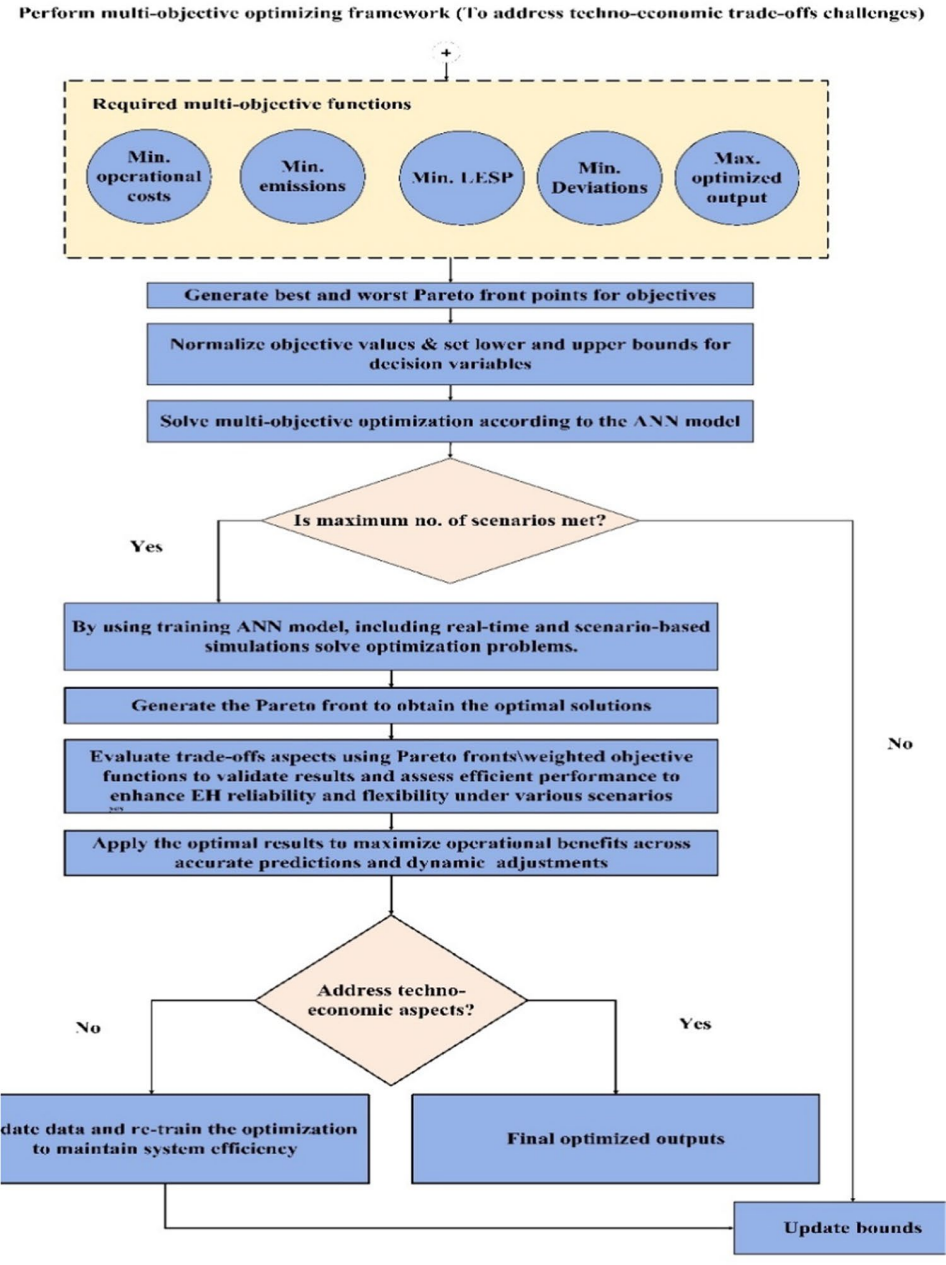
Multi-objective optimization framework.

The proposed multi-objective functions can be performed mathematically, ensuring the system’s performance and reliable operation by considering technical and environmental impacts and system constraints. The main objective functions can be classified according to the target outcome, such as maximizing system efficiency, reducing costs, minimizing pollution, or ensuring system reliability. The EH model was developed using MATLAB software within a multi-objective framework, as shown in Fig. 5. Uncertainty was addressed through stochastic modeling, while the optimization process used integration between the ANN and AL approaches to enhance the model’s predictive capabilities.

### Objective functions

The problem definition encompasses multiple objective functions, system constraints, and optimization methodologies. To ensure the solution’s applicability, the analysis must incorporate a comprehensive set of constraints, as outlined below:

**Fig. 6:**
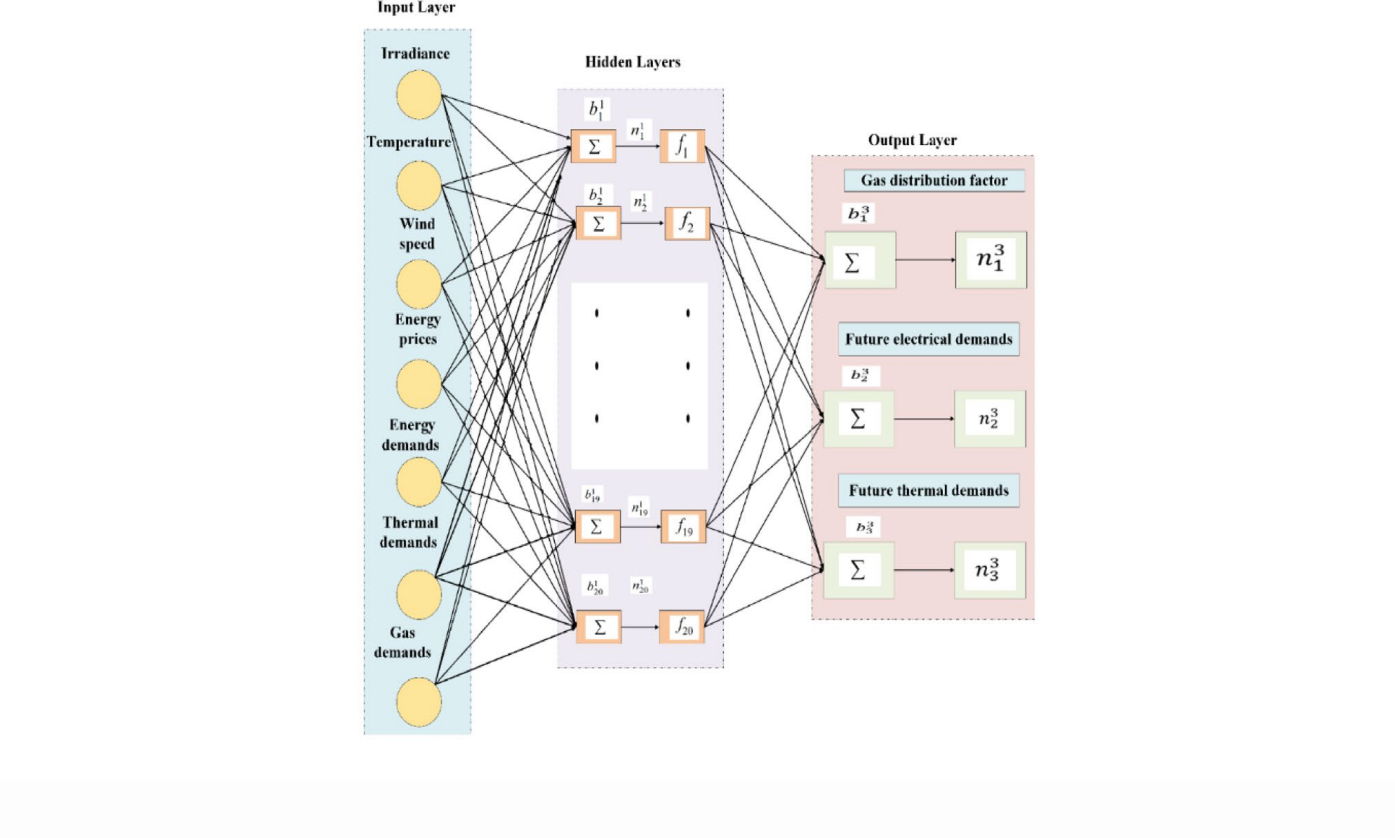
Proposed ANN-based AL structure.

#### Cost minimization

The cost-minimization objective aims to reduce the total operational costs of the EH system during power generation, distribution, and consumption. This includes minimizing expenses related to fuel, operations, maintenance, and other associated costs. Optimization considers the economic implications of various energy sources, energy storage systems, conversion technologies, and grid interactions to achieve an efficient and cost-effective energy management strategy.

**Fig. 7:**
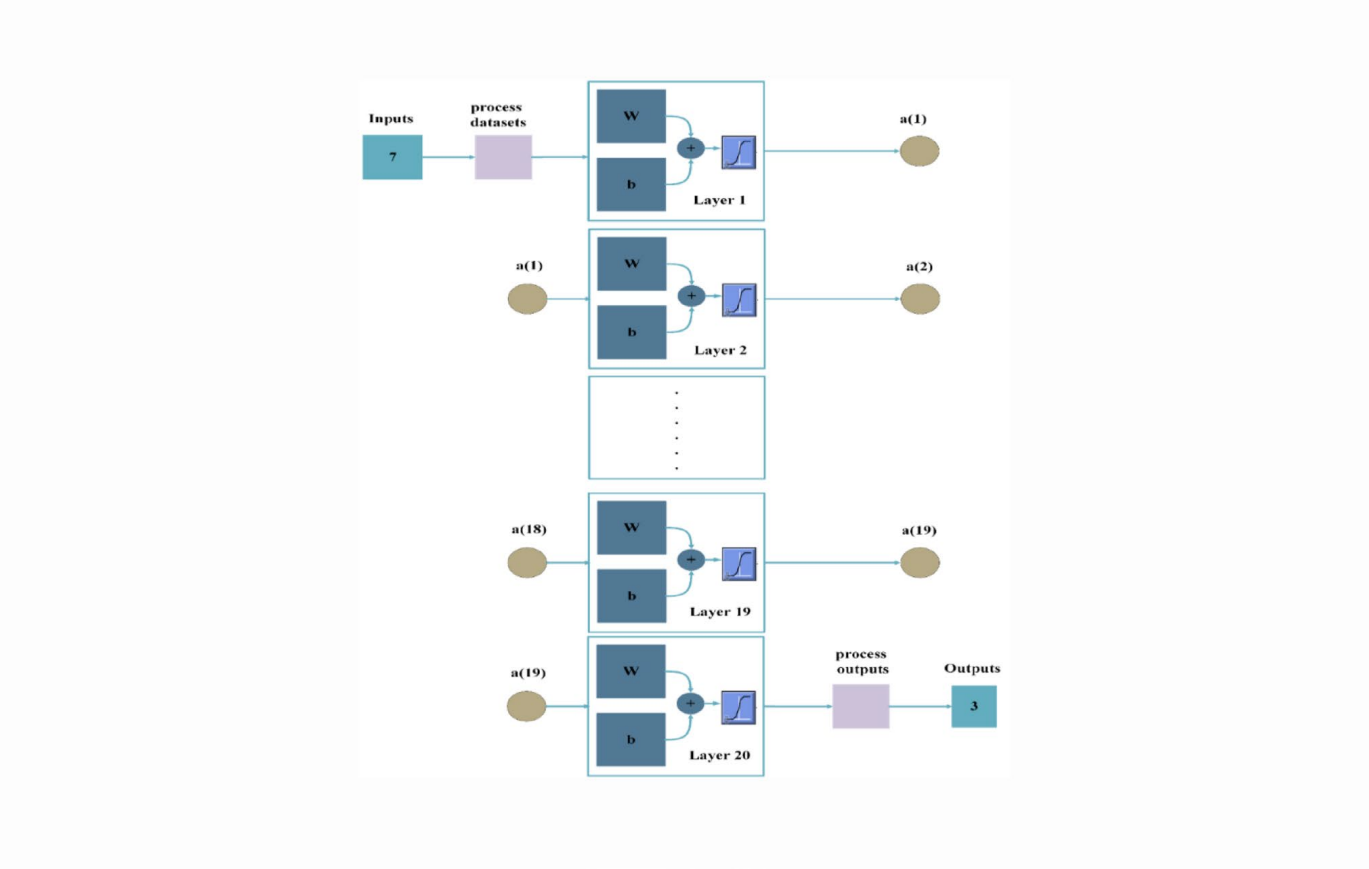
Configuration of the proposed ANN-based AL approach.

The net electrical network power $\:{P}_{E}$ generated by RESs, DGs, NG, SGs, CHP, and hydropower plants, connected to the grid, is defined as the total power output from these resources^7^ as represented by (15):

15 $$\:{P}_{E}={{P}_{wind\langle v\rangle}+{P}_{PV}+P}_{E}^{Hydro}+\:{P}_{E}^{Grid}+\:{P}_{E}^{SG}+\:{P}_{E}^{DG}+{P}_{E}^{NG}+{P}_{E}^{CHP}$$

The thermal network power $\:{Q}_{th\:}$is defined as the total output power generated by the summation of the thermal outputs of DGs, NG, SGs, and CHP units^46^ as represented by (16).

16 $$\:{Q}_{th}=\:{Q}_{th}^{NG}+\:{Q}_{th}^{SG}+{Q}_{th}^{CHP}$$

The operating cost for the electrical power $$\:{C}_{E}$$ generated by PV, WT, and hydropower plants, connected to the grid as represented by (17), where $$\:{\pi\:}^{E}$$ is the grid market price for electrical power ($/kWh):

17 $$\:{C}_{E}\left(s,t\right)=\:\:\sum\left({\pi\:}^{E}\left({{P}_{wind\langle v\rangle}+{P}_{PV}+P}_{E}^{Hydro}+\:{P}_{E}^{Grid}\right)\right)\:$$

The operation cost for the electrical and thermal power generated by DGs, NG, SGs, and CHP are $$\:{C}_{E}^{fuel}$$and $$\:{C}_{th}^{fuel}$$respectively as represented by (18) and (19), where $$\:{\pi\:}^{fuel}$$ is the fuel market price ($/kWh):

18 $$\:{C}_{E}^{fuel}\left(s,t\right)=\:\:\sum{\pi}^{fuel}\left({{x}_{DG}\:P}_{E}^{DG}+{{x}_{SG}\:P}_{E}^{SG}+{{x}_{NG}\:P}_{E}^{NG}+{{x}_{CHP}\:P}_{E}^{CHP}\right)$$

19 $$\:{C}_{th}^{fuel}=\:\:\sum\left({\pi}^{fuel}\left({{x}_{SG}\:Q}_{E}^{SG}+{{x}_{NG}\:Q}_{E}^{NG}+{{x}_{CHP}\:Q}_{E}^{CHP}\right)\right)\:\:\:\:$$

The overall operating cost $\:{C}_{O}^{E}$ for $\:EVs$ and $\:BS$^57^ and overall operating cost $\:{C}_{O}^{th}$ for thermal storage unit as in (20) and (21) respectively, illustrated as follows:

20 $$\:{C}_{O}^{E}={C}_{EVs\:}+{C}_{BS}=\:\sum\limits_{t}{\pi}^{E}\:(\:\left({P}_{ch}^{EVs}+{P}_{ch}^{BS}\right)\:+\:\left({P}_{dis}^{EVs}+\:{P}_{dis}^{BS}\right)\:)\:.\:\varDelta t$$

21 $$\:{C}_{O}^{th}=\:\sum\limits_{t}{\pi}^{fuel}\:(\:{Q}_{ch}^{th}\:+\:{Q}_{dis}^{th})\:.\:\varDelta t$$

Thus, the total cost-minimizing objective function during power generation^7^ can be expressed as in (22), where $\:s$ is the number of scenarios and $\:{\rho\:}_{S}$^46^ is the probability of these scenarios:

22 $$\:min\:{f}_{1}=\:\sum\limits_{s=1}^{S}{\rho}_{S}\sum\limits_{t=1}^{T}\left(\:{C}_{E}\left(s,t\right)+{C}_{E}^{fuel}\left(s,t\right)+{C}_{O}^{E}\left(s,t\right)+{C}_{th}^{fuel}\left(s,t\right)+\:{C}_{O}^{th}\left(s,t\right)+\sum\limits_{i}^{N}\left(\frac{{C}_{cap,i}+{C}_{O\&M,i}}{{L}_{i}}\right)\right)$$

where $\:i$ is index for each component, $\:{C}_{cap,i}$is capital cost for each unit, $\:{C}_{O\&M,i}$ is maintenance and operating cost over lifetime, and $\:{L}_{i}$ is lifetime of each component as in (23) and (24).

23 $$\:{C}_{cap,i}=\:{Capex}_{i}\:{Capacity}_{i}$$

24 $$\:{C}_{O\&M,i}=\:{Cost}_{O\&M,i}\:{Capacity}_{i}\:{L}_{i}$$

The specific parameters of each component for EH is presented as the following in Table 2.

#### Energy efficiency maximization

The objective focuses on improving the overall efficiency of the EH systems by optimizing energy conversion processes, reducing energy losses, and maximizing the integration and utilization of RESs. This optimization considers factors such as energy conversion efficiencies, demand-side management strategies, and load balancing to achieve optimal performance. The net profit^57^ is determined by the difference between the total income generated through energy sales or services provided within the EH system and the associated costs as in (25):

25 $$\:profit=\:\sum\limits_{s=1}^{S}{\rho}_{S}\sum\limits_{t=1}^{T}({Income}^{E}-\:Cost)$$

The objective function aims to reduce LESP^57^ and that obtained from a shortage in power^59^ needed to meet electrical and thermal demands according to modeled time as in (26). The first part of the equation presents a shortage in power needed to meet electrical and thermal demands, where $\:{P}_{ST}$ and $\:\:{Q}_{ST}$ are electrical and thermal power shortages.

26 $$\:{min}{f}_{2}=\sum\limits_{s=1}^{S}{\rho}_{S}\sum\limits_{t=1}^{T}\left(\frac{{P}_{ST}(s,t)}{{D}_{E}(s,t)}\right)+\left(\frac{{Q}_{ST}(s,t)}{{D}_{th}(s,t)}\right)$$

**Table 2:** EH specific parameters for each component.

PV parameters^34,58^			WT parameters^34,58^			DG parameters^34,58^			
**Rated power** $$\:{(\varvec{C}\varvec{a}\varvec{p}\varvec{a}\varvec{c}\varvec{i}\varvec{t}\varvec{y}}_{\varvec{i}}$$ **)**	200 kW		**Rated power** $$\:{(\varvec{C}\varvec{a}\varvec{p}\varvec{a}\varvec{c}\varvec{i}\varvec{t}\varvec{y}}_{\varvec{i}}$$ **)**	1kw		**Fuel cost**		0.78$/l	
**Efficiency**	16%		**Maintenance cost (** $$\:{\varvec{C}\varvec{o}\varvec{s}\varvec{t}}_{\varvec{O}\&\varvec{M},\varvec{i}}$$ **)**	10$/year		**Initial cost (** $$\:{\varvec{C}\varvec{a}\varvec{p}\varvec{e}\varvec{x}}_{\varvec{i}}\:$$ **)**		336$/year	
**Maintenance cost (** $$\:{\varvec{C}\varvec{o}\varvec{s}\varvec{t}}_{\varvec{O}\&\varvec{M},\varvec{i}}$$ **)**	5$/year					**Maintenance cost (** $$\:{\varvec{C}\varvec{o}\varvec{s}\varvec{t}}_{\varvec{O}\&\varvec{M},\varvec{i}}$$ **)**		0.05$/h	
**Lifetime (** $$\:{\varvec{L}}_{\varvec{i}}$$ **)**	20 years		**Initial cost (** $$\:{\varvec{C}\varvec{a}\varvec{p}\varvec{e}\varvec{x}}_{\varvec{i}}\:$$ **)**	1000$		**Lifetime (** $$\:{\varvec{L}}_{\varvec{i}}$$ **)**		5 years	
**Initial cost (** $$\:{\varvec{C}\varvec{a}\varvec{p}\varvec{e}\varvec{x}}_{\varvec{i}}\:$$ **)**	214.5$		**Lifetime**	20 years		**Rated power** $$\:{(\varvec{C}\varvec{a}\varvec{p}\varvec{a}\varvec{c}\varvec{i}\varvec{t}\varvec{y}}_{\varvec{i}}$$ **)**		550 kW	
**CHP parameters** ^47^			**NG parameters** ^27^			**SG parameters** ^41,47,48^			
**Rated power** $$\:{(\varvec{C}\varvec{a}\varvec{p}\varvec{a}\varvec{c}\varvec{i}\varvec{t}\varvec{y}}_{\varvec{i}}$$ **)**	550 kW		**Natural gas price**	$$\:{\pi\:}^{fuel}$$	0.06 $/$$\:{m}^{3}$$	**Rated power** $$\:{(\varvec{C}\varvec{a}\varvec{p}\varvec{a}\varvec{c}\varvec{i}\varvec{t}\varvec{y}}_{\varvec{i}}$$ **)**		1000 kW	
**Efficiency**	$$\:{\eta\:}_{E}^{CHP}$$	0.35	**Grid electricity** **price**	$$\:{\:\pi\:}^{E}$$	0.055 $/kWh	**Efficiency**		$$\:{\eta\:}_{E}^{SG}$$	0.35
	$$\:{\eta\:}_{th}^{CHP}\:$$	0.40						$$\:{\eta\:}_{th}^{SG}\:$$	0.40
**Maintenance cost (** $$\:{\varvec{C}\varvec{o}\varvec{s}\varvec{t}}_{\varvec{O}\&\varvec{M},\varvec{i}}$$ **)**	40$/kw		**Efficiency**	$$\:{\eta\:}_{E}^{CHP}$$	0.35	**Initial cost (** $$\:{\varvec{C}\varvec{a}\varvec{p}\varvec{e}\varvec{x}}_{\varvec{i}}\:$$ **)**		90 $	
**Lifetime (** $$\:{\varvec{L}}_{\varvec{i}}$$ **)**	20 years			$$\:{\eta\:}_{th}^{CHP}\:$$	0.40	**Maintenance cost (** $$\:{\varvec{C}\varvec{o}\varvec{s}\varvec{t}}_{\varvec{O}\&\varvec{M},\varvec{i}}$$ **)**		40$/kw	
**Initial cost (** $$\:{\varvec{C}\varvec{a}\varvec{p}\varvec{e}\varvec{x}}_{\varvec{i}}\:$$ **)**	1200$		**Lifetime (** $$\:{\varvec{L}}_{\varvec{i}}$$ **)**	20 years		**Lifetime (** $$\:{\varvec{L}}_{\varvec{i}}$$ **)**		20 years	
**BS parameters** ^34,44,50^				**EV parameters** ^4,14,57^					
**Rated power** $$\:{(\varvec{C}\varvec{a}\varvec{p}\varvec{a}\varvec{c}\varvec{i}\varvec{t}\varvec{y}}_{\varvec{i}}$$ **)**	200 kWh			$$\:{\varvec{P}}_{\varvec{c}\varvec{h}}^{\varvec{E}\varvec{V}}$$		200 kW			
**Maintenance cost (** $$\:{\varvec{C}\varvec{o}\varvec{s}\varvec{t}}_{\varvec{O}\&\varvec{M},\varvec{i}}$$ **)**	1800 $/year			$$\:{\varvec{P}}_{\varvec{d}\varvec{i}\varvec{s}}^{\varvec{E}\varvec{V}}$$		200 kW			
**Lifetime (** $$\:{\varvec{L}}_{\varvec{i}}$$ **)**	10 years			$$\:{\varvec{\eta\:}}_{\varvec{c}\varvec{h}}^{\varvec{E}\varvec{V}}$$		0.9			
**Initial cost (** $$\:{\varvec{C}\varvec{a}\varvec{p}\varvec{e}\varvec{x}}_{\varvec{i}}\:$$ **)**	47,400			$$\:{\varvec{\eta\:}}_{\varvec{d}\varvec{i}\varvec{s}}^{\varvec{E}\varvec{V}}$$		0.9			

#### Pollution minimization

This objective aims to minimize carbon emissions arising from energy generation and consumption. It focuses on optimizing the utilization of RESs, alleviating reliance on fossil fuels, and implementing energy efficiency measures. Additionally, it considers the carbon intensity of various energy sources, prioritizing the integration of low-carbon by regulating GDFs and carbon-free alternatives to achieve a more sustainable and environmentally responsible energy system^7^ where $\:{E}^{{co}_{2}}$ represents the carbon emission rate (kg/kWh). The emission rates^43^ for the electrical and thermal power generated by DGs, NG, SGs, and CHP are $\:{E}_{E}^{fuel}$ and $\:{E}_{th}^{fuel}$ respectively as represented by (27) and (28):

27 $$\:{E}_{E}^{fuel}=\:\:\sum{E}^{{co}_{2}}\:\left({{x}_{DG}\:P}_{E}^{DG}+{{x}_{SG}\:P}_{E}^{SG}+{{x}_{NG}\:P}_{E}^{NG}+{{x}_{CHP}\:P}_{E}^{CHP}\right)$$

28 $$\:{E}_{th}^{fuel}=\:\:\sum\left({E}^{{co}_{2}}\:\left({{x}_{SG}\:Q}_{th}^{SG}+{{x}_{NG}\:Q}_{th}^{NG}+{{x}_{CHP}\:Q}_{th}^{CHP}\right)\right)\:\:\:\:$$

This objective seeks to minimize $\:{CO}_{2}$ emissions within the EH system, as a critical measure to address pressing environmental challenges and promote sustainable energy practices as in (29):

29 $$\:{min}{f}_{3}=\:\sum\limits_{s=1}^{S}{\rho}_{S}\sum\limits_{t=1}^{T}({E}_{E}^{fuel}\left(s,t\right)+{E}_{th}^{fuel}\left(s,t\right))$$

#### Optimal energy utilization

This objective seeks to enhance the integration and utilization of RESs within the EH systems. This involves optimizing the scheduling and dispatch of RES-generated power, ensuring coordination with BS units, managing the inherent intermittency of RESs, and improving the predictability of sudden fluctuations in their output. The approach incorporates RES output as well as power contributions from DGs, SGs, NG, CHP units, BS, and EVs capacities, while accounting for demand patterns to maximize RES penetration. The associated objective function minimizes the deviation between predicted optimal and actual levels of required electrical and thermal demands, as in (30). This framework can be formulated as a mathematical optimization problem, designed to identify optimal configurations and parameter values within the EH system to achieve the desired objectives of efficiency, reliability, and sustainability^7^.

30 $$\:{min}{f}_{4}=\:\sum\limits_{s=1}^{S}{\rho}_{S}\sum\limits_{t=1}^{T}(\left|{\widehat{D}}_{E}\left(s,t\right)-{D}_{E}^{actual}\right|+\left|{\widehat{D}}_{th}\left(s,t\right)-{D}_{th}^{actual}\right|)$$

The actual levels for required electrical and thermal demands are $\:{D}_{E}^{actual}$ and $\:{D}_{th}^{actual}$ respectively as represented by (31) and (32):

31 $$\:{D}_{E}^{actual}=\frac{\sum_{t=1}^{T}{D}_{E}}{T}$$

32 $$\:{D}_{th}^{actual}=\frac{\sum_{t=1}^{T}{D}_{th}}{T}$$

### System constraints

To effectively address the techno-economic challenges associated with EH, the proposed approach must incorporate a comprehensive set of constraints to ensure optimal solutions are achieved:

#### Energy balance constraints

Energy balance constraints are designed to ensure that the generated power satisfies the required demand under each scenario. Specifically, these constraints address the balance for electrical and thermal power in EHs, respectively as given in (33) and (34):

33 $$\:{P}_{E}\left(s,t\right)+{P}_{ST}\left(s,t\right)+{P}_{dis}^{EVs}\left(s,t\right)+\:{P}_{dis}^{BS}\left(s,t\right)={D}_{E}\left(s,t\right)+{P}_{ch}^{EVs}\left(s,t\right)+{P}_{ch}^{BS}\left(s,t\right)$$

34 $$\:{Q}_{th}\left(s,t\right)+{Q}_{ST}(s,t)+\:{Q}_{dis}^{th}\left(s,t\right)=\:{D}_{th}\left(s,t\right)+{Q}_{ch}^{th}\left(s,t\right)$$

#### Energy limitations

Energy limitation constraints^47^ define the minimum and maximum power boundaries for DGs^7^ as in (35), SGs as in (36), CHP as in (37), and NG as in (38) modules. The limitations for thermal storage units can be obtained as in (42).

35 $$\:{P}_{DG}^{min}\:\le\:\:{P}_{E}^{DG}\left(s,t\right)\:\le\:\:{P}_{DG}^{max}$$

36 $$\:{T}_{SG}^{min}\:\le\:\:{P}_{E}^{SG}\left(s,t\right)\:\le\:\:{T}_{SG}^{max}$$

37 $$\:{P}_{CHP}^{min}\:\le\:\:{P}_{E}^{CHP}\left(s,t\right)\:\le\:\:{P}_{CHP}^{max}\:\:\:$$

38 $$\:{P}_{NG}^{min}\:\le\:\:{P}_{E}^{NG}\left(s,t\right)\:\le\:\:{P}_{NG}^{max}\:\:\:$$

39 $$\:{Q}_{th}^{min}\:\le\:\:{Q}_{th}\left(s,t\right)\:\le\:\:{Q}_{th}^{max}$$

The constraints for BS units for discharging and charging states^57^ can be obtained, where $\:{\mu\:}_{dis}^{BS}$ and $\:{\mu\:}_{ch}^{BS}$are binary values^7^ for BS in discharging and charging rates as in (40) and (41) respectively.

40 $$\:\frac{{P}_{dis}^{BS}\left(s,t\right)}{{\eta\:}_{dis}^{BS}}\:\le\:\:{P}_{dis}^{BS\_Max}.{\mu\:}_{dis}^{BS}\:\left(s,t\right)\:$$

41 $$\:{P}_{ch}^{BS}*\:{\eta\:}_{ch}^{BS}\:\le\:\:{P}_{ch}^{BS\_Max}.\:{\mu\:}_{ch}^{BS}\:\left(s,t\right)$$

The constraints for EVs for discharging and charging states can be obtained, where $\:{\mu\:}_{dis}^{EV}$ and $\:{\mu\:}_{ch}^{EV}$are binary values for EVs’ discharging and charging rates, as in (42) and (43), respectively.

42 $$\:\frac{{P}_{dis}^{EV}\left(s,t\right)}{{\eta\:}_{dis}^{EV}}\:\le\:\:{P}_{dis}^{EV\_Max}.{\mu\:}_{dis}^{EV}\:\left(s,t\right)\:$$

43 $$\:{P}_{ch}^{EV}*\:{\eta\:}_{ch}^{BS}\:\le\:\:{P}_{ch}^{EV\_Max}.\:{\mu\:}_{ch}^{EV}\:\left(s,t\right)$$

The constraints for thermal storage units for discharging and charging states^57^ can be obtained, where $\:{\mu\:}_{dis}^{th}$ and $\:{\mu\:}_{ch}^{th}$are binary values for it in discharging and charging rates, as in (44) and (45), respectively.

44 $$\:\frac{{Q}_{dis}^{th}\left(s,t\right)}{{\eta\:}_{dis}^{th}}\:\le\:\:{Q}_{dis}^{th\_Max}.{\mu\:}_{dis}^{th}\:\left(s,t\right)\:$$

45 $$\:{Q}_{ch}^{th}*\:{\eta\:}_{ch}^{th}\:\le\:\:{Q}_{ch}^{th\_Max}.\:{\mu\:}_{ch}^{th}\:\left(s,t\right)$$

Thus, BS and thermal storage units are not capable of charging or discharging at the same time, these binary value limitations^7^ for BS are represented by (46), limitations for EVs as in (47), and limitations for thermal storage units as in (48), respectively.

46 $$\:\:{\mu\:}_{dis}^{BS}\:\left(s,t\right)+{\mu\:}_{ch}^{BS}\:\left(s,t\right)\le\:1$$

47 $$\:\:{\mu\:}_{dis}^{EV}\:\:\left(s,t\right)+{\mu\:}_{ch}^{EV}\left(s,t\right)\le\:1$$

48 $$\:\:{\mu\:}_{dis}^{th}\:\left(s,t\right)+{\mu\:}_{ch}^{th}\left(s,t\right)\le\:1$$

The constraints for electrical and thermal power shortages^7^ are set to ensure that the demand requirements in EH are met, as performed in (49) and (50), where $\:{{\upmu\:}}_{PST}^{E}$ and $\:{{\upmu\:}}_{PST}^{th}$ represents binary values for shortage in electrical and thermal power.

49 $$\:0\:\le\:\:{P}_{ST}\left(s,t\right)\:\le\:{D}_{E}\left(s,t\right)\:.\:\:{\mu\:}_{PST}^{E}\left(s,t\right)$$

50 $$\:0\:\le\:\:{Q}_{ST}(s,t)\:\le\:{D}_{th}\left(s,t\right)\:.\:\:{\mu\:}_{PST}^{th}\left(s,t\right)$$

#### Battery and thermal storage unit technical constraints

The technical constraints^4^ according to energy dynamics limitation for BS as in (51), EVs as in (52), and thermal storage unit as in (53), respectively.

51 $$\:{E}_{BS}^{Min}\le\:{E}_{BS}\left(s,t\right)\le\:{E}_{BS}^{Min}$$

52 $$\:{E}_{EV}^{Min}\le\:{E}_{EV}\left(s,t\right)\le\:{E}_{EV}^{Min}$$

53 $$\:{E}_{th}^{min}\le\:\:{E}_{th}\left(s,t,tss\right)\le\:{E}_{th}^{max}$$

The energy dynamics limitation^4^ for BS can be obtained as in (54), EVs as in (55), and thermal storage unit as in (56), respectively.

54 $$\:{E}_{BS}\left(s,t\right)=\:{E}_{BS}\left(s,t-1\right)+\left[\frac{{P}_{dis}^{BS}\:\left(s,t\right)}{{\eta\:}_{dis}^{BS}}-\:{P}_{ch}^{BS}\left(s,t\right)\:.\:\:{\eta\:}_{ch}^{BS}\right]\:$$

55 $$\:{E}_{EV}\left(s,t\right)=\:{E}_{EV}\left(s,t-1\right)+\left[\frac{{P}_{dis}^{EV}\:\left(s,t\right)}{{\eta\:}_{dis}^{EV}}-\:{P}_{ch}^{EV}\left(s,t\right)\:.\:\:{\eta\:}_{ch}^{EV}\right]$$

56 $$\:{E}_{th}\left(s,t\right)=\:{E}_{th}\left(s,t-1\right)+\left[\frac{{P}_{dis}^{th}\:\left(s,t\right)}{{\eta\:}_{dis}^{th}}-\:{P}_{ch}^{th}\left(s,t\right)\:.\:\:{\eta\:}_{ch}^{th}\right]$$

#### Gas distribution constraints

The technical constraints for regulating GDFs between DGs, SGs, NG, and CHP units are governed by the parameter efficiency for each generating unit, which is defined as the minimum allowable efficiency for total output power generated by these units. The technical constraint^7^ for the electrical network is as in (57), and the constraint for the thermal network is as in (58).

57 $$\:{{{P}_{wind\langle v\rangle}+{P}_{PV}+P}_{E}^{Hydro}+\:{P}_{E}^{Grid}+{P}_{E}^{NG}{x}_{NG}+{P}_{E}^{DG}{x}_{DG}+{P}_{E}^{SG}{x}_{SG}+{P}_{E}^{CHP}{x}_{CHP}\:\ge\:\:D}_{E}$$

58 $$\:{{Q}_{th}^{NG}{x}_{NG}++{Q}_{th}^{SG}{x}_{SG}+{Q}_{th}^{CHP}{x}_{CHP}\:\ge\:\:D}_{th}\:$$

The limitation of parameter efficiency for DGs^47^ SGs, NG^27^ and CHP^48^ units can be expressed as in (59):

59 $$\:\frac{\sum_{i=1}^{x}{x}_{i}{\eta\:}_{i}}{\sum{x}_{i}}\:\ge\:\:{\eta\:}_{i}^{Min}\:,\:\:\:\:\:{x}_{NG}+\:{x}_{DG}+{x}_{SG}+\:{x}_{CHP}=1$$.

## Artificial neural network

**Fig. 8:**
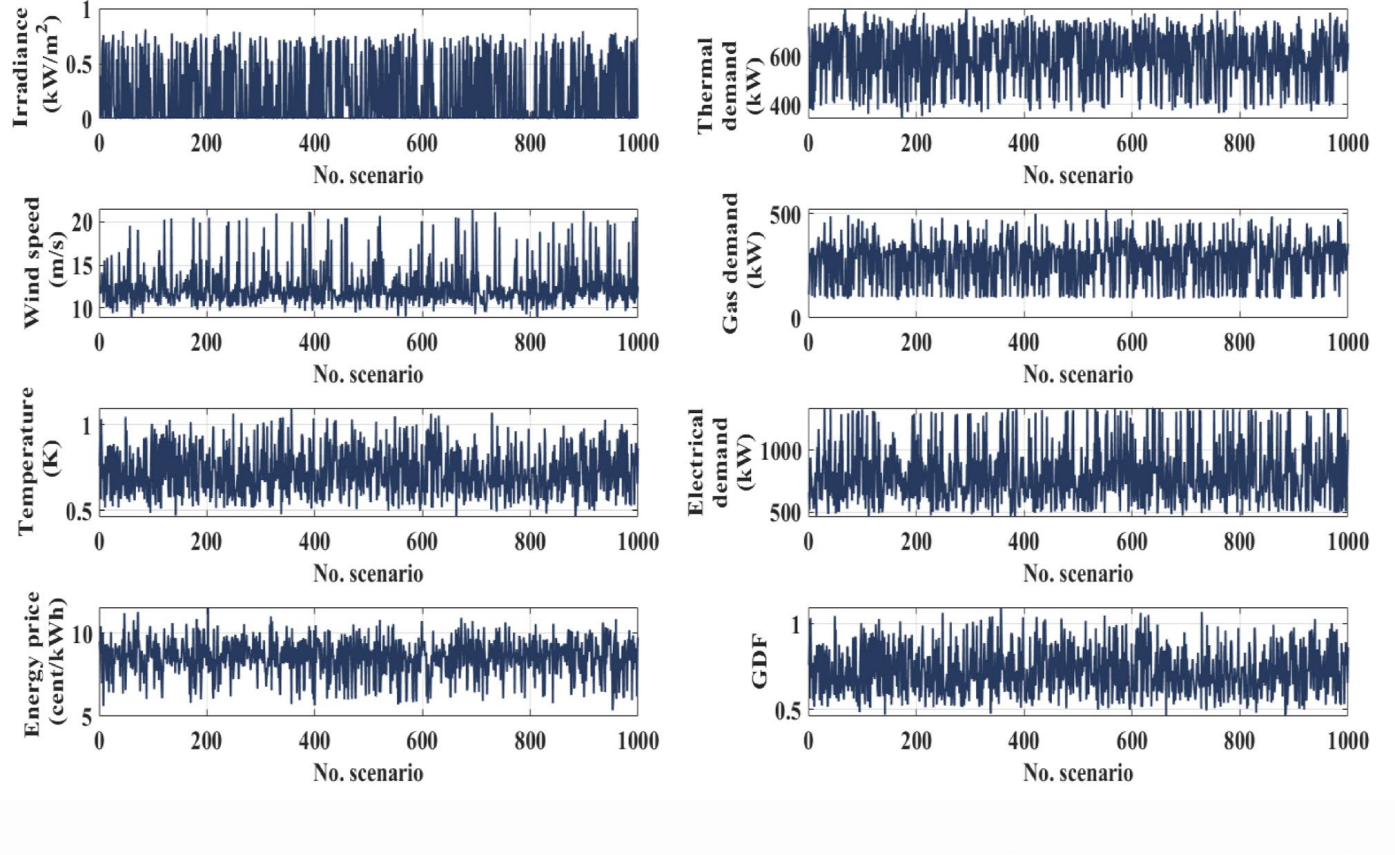
Monte Carlo simulated dataset scenarios: irradiance, temperature wind speed, energy prices, heat, energy and gas demands, and GDF.

**Fig. 9:**
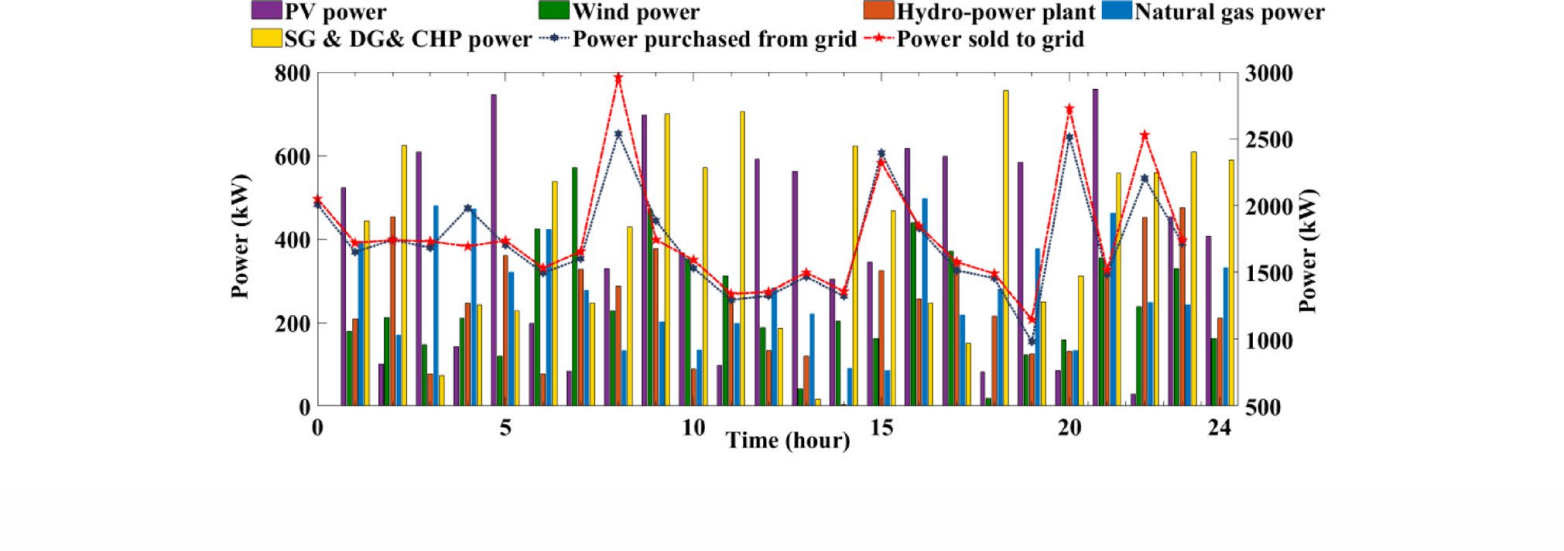
Hourly output generated power and grid interaction dynamics for managing peak demand periods.

This study employs a backpropagation algorithm to implement a multilayer perceptron (MLP) with a single hidden layer in a feed-forward architecture^22^. The proposed ANN comprises three layers: input, hidden, and output. The input layer includes seven neurons, $\:{x}_{i}{\epsilon}\:{u}^{d}$, where $\:u$ represents $\:{matrix}_{1x7}$, $\:d$ represents set dimension for the input dataset, as in (60). The hidden layer contains 20 neurons, while the output layer produces a three-dimensional vector, where $\:y$ represents ANN model output, representing GDFs, future electrical demands $\:{\widehat{D}}_{E}$, and future thermal demands $\:{\widehat{D}}_{th}$, as in (61). A compact network architecture is adopted to reduce computational complexity and accelerate training, making it well-suited for real-time energy hub applications requiring fast and efficient decision-making. The proposed neuron model consists of interconnected links, referred to as synapses, where $\:{w}_{ki}$ represents the connection weight of the $\:{i}_{th}\:$input unit. Each weight is multiplied by its corresponding input $\:{x}_{i}$, and the sum is adjusted by a bias term $\:{b}_{ki}$^60^ to control the output summation, which is important for decreasing or raising the output summation,$\:\:{n}_{k}^{i}$. To limit the amplitude range of the output signals to a finite value, an activation function $\:{f}_{i}\left(n\right)$, resulting in the output signals^60^ $\:{a}_{k}^{i}$ to reduce its amplitude range to a finite value. The proposed ANN structure highlights the model’s systematic design and functionality in effectively addressing the targeted problems as in Figs. 8 and 9.

60 $$\:u=[irradiance;\:temperature;\:wind\:speed;\:energy\:prices;\:{D}_{E};\:{D}_{th};\:gas\:demands\:]$$

61 $$\:y=[GDF;\:{\widehat{D}}_{E};\:{\widehat{D}}_{th}]$$

Dataset preparation plays a vital role in structuring input-target pairs, such as energy demand characteristics matched with system performance metrics. For EHs, this involves aligning energy and gas demand features with operational performance indicators to build a robust foundation for modeling and optimizing system dispatch. The framework accounts for critical factors influencing energy generation and consumption, including temporal variations, weather conditions, energy price fluctuations, and historical usage trends.

These input-target pairs are essential for predicting optimal GDFs across generation units and accurately forecasting future electrical and thermal demands. Additionally, system performance metrics, such as energy efficiency and reliability indices, serve as target outputs, further guiding the optimization of EH operations. The dataset undergoes $\:Min-Max$ normalization^16^ as expressed in (62), to ensure feature compatibility, numerical stability, and improved convergence rates during training.

62 $$\:\stackrel{\prime }{{x}_{i}}=\:\frac{{x}_{i}-min\left(u\right)}{{max}\left(u\right)-min\left(u\right)}$$

The proposed dataset undergoes a normalization process to scale all input features within a defined range, facilitating efficient pattern recognition by the ANN model. It is then divided into training, validation, and testing sets to ensure effective model training, parameter tuning, and performance assessment. During forward propagation, the input features pass through multiple ANN layers, where each neuron performs a weighted summation, adds bias terms, and applies activation functions^60^. The resulting outputs are passed to the output layer, the output layer, where the model generates predictions. These predictions are then evaluated against actual target values using a loss function to quantify the prediction error. For regression tasks, the mean squared error (MSE)^55^ as expressed in (63), is commonly employed, while cross-entropy loss is typically used for classification problems.

63 $$\:MSE=\frac{1}{N}\sum\limits_{i=1}^{N}{({y}_{i}-y)}^{2}$$

Backpropagation is a method used to adjust ANN parameters and update weights and biases iteratively to minimize errors. It uses the chain rule of differentiation to compute gradients of the loss function, ensuring precise parameter updates and improved model accuracy. Stochastic gradient descent (SGD) is used to minimize the loss function. The optimization process updates model parameters using mini batches of randomly selected data, enhancing learning capabilities for large-scale datasets. The training dataset is processed multiple times until predefined criteria are met.

To ensure the proposed approach’s robustness under various uncertainties, the discrepancy between the model’s optimal predictions and the actual data is assessed across different scenarios. The neural network’s predictive accuracy is evaluated using performance metrics such as the mean relative error (MRE), as in (64), and normalized root mean square error (NRMSE)^55^ as in (65). In this study, the NRMSE was utilized to validate the model’s accuracy and to assess the similarity between optimal predicted and actual values. Ideally, an NRMSE value of 0 represents the highest model performance, indicating perfect agreement between optimal predictions and actual outcomes.

64 $$\:RMSE=\:\sqrt{MSE}\:=\:\sqrt{\frac{1}{N}\sum\nolimits_{i=1}^{N}{({y}_{i}-y)}^{2}}$$

65 $$\:NRMSE=\:\frac{RMSE}{mean\left(y\right)}$$

The proposed ANN-based prediction model is designed within a multi-objective optimization framework to deliver high precision in both training and forecasting tasks. Recognizing that inaccuracies in input parameter estimation can compromise model performance, an AL approach is employed to improve input computation. The model undergoes an iterative optimization cycle aimed at minimizing prediction errors, supported by comprehensive validation and testing to ensure robustness and dependability. The training process includes structured steps such as dataset preprocessing, forward propagation, loss evaluation, backpropagation, and dynamic weight adjustments, collectively enhancing the model’s accuracy and adaptability. To improve energy demand prediction and reduce operational costs in EHs, this study incorporates an ANN-based AL approach within a multi-objective optimization framework that accounts for uncertainties, operational constraints, load fluctuations, energy price volatility, and GDFs. The process begins with loading EH-related datasets and defining uncertainty parameters, followed by generating potential scenarios and identifying optimization functions based on sustainability metrics. The є-constraint method is used to solve the objective functions and determine optimal decision variables. An active learning strategy is then applied to select and label the most informative data points, updating the dataset and retraining the ANN to improve predictive accuracy. This iterative cycle continues until the model achieves the desired performance. By integrating optimization with adaptive learning, the proposed framework enhances prediction accuracy, improves system reliability, and achieves efficient EH operation using fewer labeled data samples.

## Numerical results and their discussion

The paper explores the trade-offs of optimizing EHs management, aiming to balance cost-effectiveness, system reliability, and environmental sustainability. It uses a multi-objective functions model to consider uncertainty factors from weather variability^60^ energy price fluctuations, unplanned outages, and dynamic demand changes. The study simulated 1,000 scenarios using historical datasets to predict future electrical and thermal demands and GDFs. The ANN-based AL approach improves the sustainability of EH operation by enhancing adaptability to nonlinear interactions, leveraging advanced learning from dynamic system behaviors, and iteratively selecting and updating informative datasets for training. The integration of AL approach improves the predictive and optimization capabilities of the ANNs, leading to reduced costs and improved system efficiency. The ANN-based AL framework ensures reliable predictions and minimizes the need for extensive labeled data, making it a crucial tool for optimizing system operations by addressing uncertainties and delivering reliable, efficient, and sustainable energy management solutions.

The paper presents numerical results and analysis demonstrating the effectiveness of an ANN-based AL framework in improving EH performance, system reliability, and energy efficiency, while finding optimal solutions for techno-economic trade-offs. It also discusses the influence of uncertainty on multi-objective optimization outcomes. The proposed EH system, depicted in Fig. 2, is developed to simultaneously reduce operational costs and emissions and improve system reliability. Its performance is assessed through three case studies conducted over a 24-hour period, in accordance with the formulated multi-objective optimization framework and the associated operational constraints. The entire system is implemented in MATLAB (version 2023b), facilitating accurate simulation and comprehensive evaluation of the EH’s behavior under various operational scenarios. The ANN-based AL approach improves EH performance by leveraging adaptive learning capabilities to make accurate predictions and dynamic adjustments. Iterative training on informative datasets enhances predictive accuracy, ensuring robust decision-making under uncertain operating conditions. This evaluation considers energy constraints to ensure feasible and sustainable solutions while optimizing system performance.

Although the integration between ANN and the AL approach with uncertainty modeling demonstrates high accuracy, they are sensitive to the quality and variety of the initial training data, which could affect early decision accuracy under uncertainty. The repetitive nature of active learning adds computational complexity, which may restrict real-time applicability in large-scale systems even as it lessens the effort required to categorize data. Furthermore, in extremely stochastic contexts, the ANN’s capacity for generalization may wane unless it is supplemented with reliable methods like ensemble or probabilistic modeling. The irradiance^46^ temperature, wind speed^46^ energy price data^43^ along with thermal, gas, and electrical demand profiles thermal, gas and electrical demand profiles^43^ were used to generate 1000 scenarios via Monte Carlo Simulation. This approach incorporates uncertainty analysis to enhance the robustness of the artificial neural network (ANN) by embedding variability and stochastic behavior into the input data. The generated scenarios, illustrated in Fig. 8, are seamlessly integrated into the modeling framework and serve as a critical training dataset during the learning phase.

**Fig. 10:**
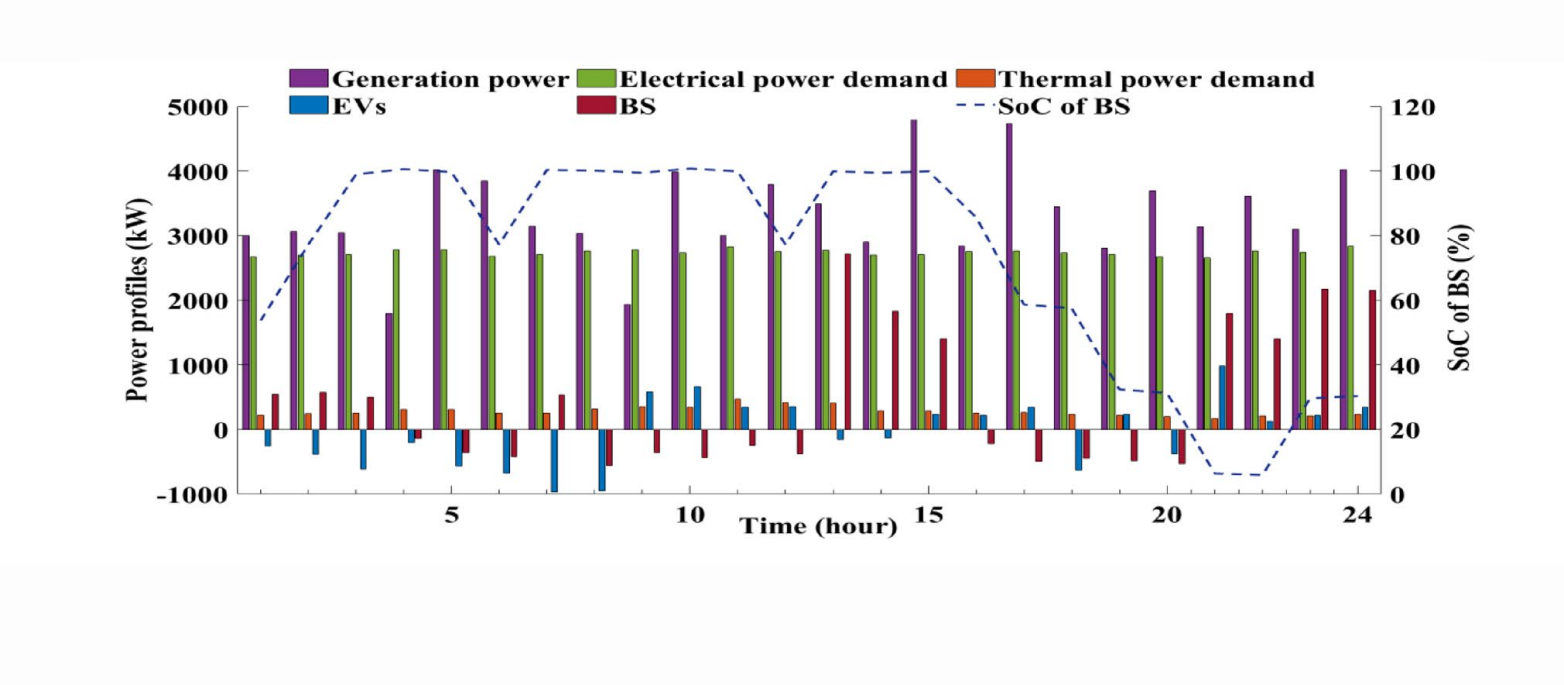
Hourly power profiles and SoC of the BS over 24 h, capturing key interactions among generation power, electrical power demand, thermal power demand, and the contributions of EVs within the system.

The proposed EH system is designed to effectively meet both electrical and thermal energy demands. Furthermore, the integration of grid-connected electric vehicles (EVs) and battery storage (BS) systems significantly enhances the system’s operational flexibility and overall reliability. This comprehensive evaluation underscores the resilience and adaptability of the proposed framework, demonstrating its capability to optimize energy hub performance across a diverse range of operating conditions and under uncertainty. The results highlight the system’s strength in accommodating dynamic energy profiles while maintaining efficient, reliable, and sustainable operation. Figure 9 presents the dynamics of output power generation and grid interaction during peak demand periods, highlighting the system’s operational efficiency and adaptability. Figure 10 depicts the 24-hour power trajectories and state of charge (SoC) of the BS, effectively capturing the interdependencies among generation output, electrical and thermal energy demands, and the contributions of EVs within the EH. Throughout the day, generation consistently surpasses electrical demand, with observed fluctuations reflecting the system’s responsive behavior in maintaining real-time supply-demand equilibrium. The SoC profile illustrated by the dashed line reveals the charging and discharging cycles of the BS, underscoring its vital function in reinforcing the system’s stability and operational flexibility.

These findings emphasize the synergistic integration of BS and EVs in satisfying energy requirements and enhancing power management. The ability to consistently meet or exceed demand signifies the robustness and reliability of the EH system. Concurrently, BS and EVs play instrumental roles in mitigating volatility and buffering supply irregularities. Nevertheless, the analysis also uncovers notable challenges in regulating EV power consumption and managing SoC dynamics during peak load intervals. These complexities underscore the need for sophisticated optimization frameworks to uphold system efficiency under variable and high-demand scenarios.

Ultimately, the evaluation affirms the effectiveness of the artificial neural network (ANN) combined with active learning (AL) methodology in augmenting the operational flexibility of the EH. The approach demonstrates strong capability in maintaining dependable performance amid peak demand conditions while adeptly navigating complex, time-varying operational landscapes, ensuring the energy hub’s stable and optimized functionality.

The proposed methodology is assessed through three distinct case studies, each designed to evaluate different aspects of the energy hub system under uncertainty:

### • Case 1

Assesses system performance using a single-objective optimization approach while accounting for operational uncertainties.

### • Case 2

Evaluates the system under a multi-objective optimization framework, incorporating uncertainty into the decision-making process.

### • Case 3

Implements the ANN-based AL approach within the multi-objective optimization framework under uncertainty, aiming to enhance adaptability and predictive performance.

The performance is highly dependent on the appropriate selection of parameter values, as poor tuning can significantly impair effectiveness. To ensure optimal outcomes, extensive parameter tuning was performed. This involved a comprehensive range of test scenarios and multiple iterations to evaluate the impact of different configurations. Through systematic experimentation, the most effective parameter settings were identified. For clarity and completeness, the revised version includes a summary table outlining the final selected parameters based on these evaluations.

**Table 3:** Algorithms parameters used in the simulation.

Algorithm	forecasting problem		Uncertainty modelling		Objective function framework	
**Case** **1**	Hidden layer size	20	-		Min. Cost, Min. emissions, Min. LESP, Min. deviation between actual and optimal levels of energy demands as a single objective function	
	No. neurons	10				
	No. epochs	1000				
	$\:{iter\:}^{\text{m}\text{a}\text{x}}$	100				
	Learning rate	0.1000				
	Batch size	20				
	Train function	trainlm				
	Goal error	1$\:{e}^{-4}$				
	Train ratio	0.7				
	Validation ratio	0.15				
	Test ratio	0.15				
	RMSE	19.0333				
**Case** **2**	Hidden layer size	20	Time interval	24	Obj. function	Min. Cost, Min. emissions, Min. LESP, Min. deviation between actual and optimal levels of energy demands as a multi-objective function
	No. neurons	10				
	No. epochs	1000			Population size	100
	$\:{iter\:}^{\text{m}\text{a}\text{x}}$	100				
	Learning rate	0.1000			Max. generations	200
	Batch size	20				
	Train function	trainlm	$\:{iter\:}^{\text{m}\text{a}\text{x}}$	1000	$\:{l}_{b}$	$\:\left[0\:0\:35\:6.8\:2\right]$
	Goal error	1$\:{e}^{-4}$			$\:{u}_{b}$	$\:\left[0.6\:0.4\:40\:7.4\:5\right]$
	Train ratio	0.7			Crossover fraction	0.6
	Validation ratio	0.15				
	Test ratio	0.15			Pareto fraction	0.3
	RMSE	0.2828				
**Case** **3**	Hidden layer size	[5 30]	Time interval	24	Obj. function	Min. Cost, Min. emissions, Min. LESP, Min. deviation between actual and optimal levels of energy demands as a multi-objective function
	no. epochs	[100 1000]				
	train function	trainlm			Population size	100
	Query budget	50				
	$\:{iter\:}^{\text{m}\text{a}\text{x}}$	100			Max. generations	200
	Best hidden layer size	30 neurons	Num. scenarios	1000		
	Training goal	$\:2.72\text{*}{10\:}^{-6}$			$\:{l}_{b}$	$\:Varied$
	Training epoch	647	normrnd	(Mean, std)	$\:{u}_{b}$	$\:\text{V}\text{a}\text{r}\text{i}\text{e}\text{d}$
	Best RMSE	0.2356				

**Fig. 11:**
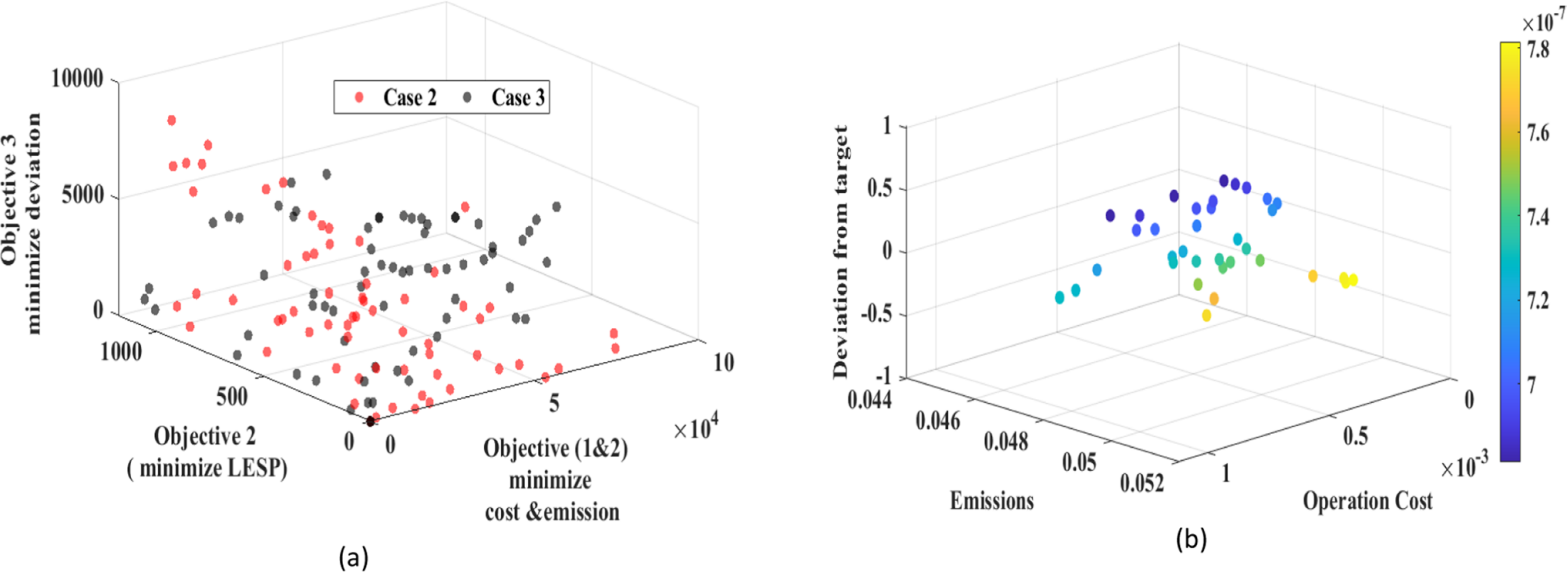
Pareto front analysis for multi-objective framework: **(a)** performance of two optimization scenarios, **(b)** 3D Pareto front projection for case 3.

**Fig. 12:**
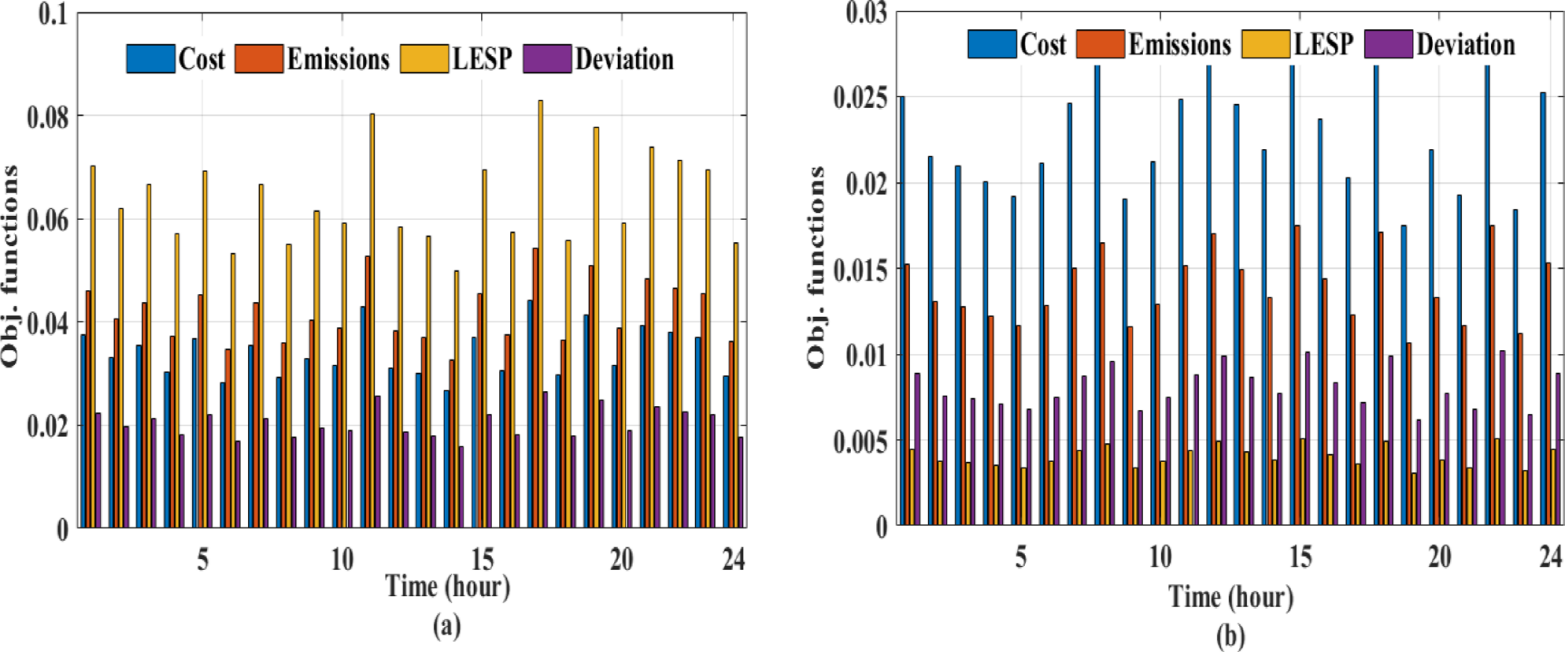
Multi-objective functions performance with uncertainty: **(a)** Case 2, **(b)** Case 3.

The multi-objective analysis depicted in Fig. 11 highlights the effectiveness of the proposed optimization framework in reconciling competing objectives, such as the minimization of operational cost, environmental emissions, and deviations from target load conditions. Figure 11(a) offers a comparative evaluation between Case 2 and Case 3, shedding light on the relative performance of each strategy. Case 3 exhibits a denser aggregation of solutions in the lower-left quadrant of the deviation and LESP axes, reflecting a higher degree of robustness in maintaining operational targets while minimizing environmental impact. In contrast, Case 2 displays a more dispersed solution space, with a noticeable presence of high-deviation outcomes, signaling reduced reliability under similar conditions. In addition, Fig. 11(b), the generated Pareto front, reveals a tightly clustered distribution of solutions along the cost and emission axis, with most configurations exhibiting minimal deviation from target values. This distribution indicates a high degree of convergence toward a well-balanced operational regime. The color gradient mapped onto the performance metric further underscores this coherence, revealing that lower-deviation solutions are frequently associated with more favorable cost and emission outcomes—demonstrating a strong alignment among the competing objectives within the optimization process. It ensured the superior performance of Case 3 in achieving a more favorable trade-off across the triad of objectives. Such insights align with core principles in evolutionary multi-objective optimization, where solution quality is assessed based not only on convergence toward the Pareto front but also on the diversity and dominance of solutions across the objective target. Figure 12 showcases the most optimal outcomes derived from the multi-objective optimization framework for both Case 2 and Case 3, providing a comparative assessment of their effectiveness in addressing complex techno-economic challenges. The ANN-based AL methodology, when integrated with uncertainty modeling, exhibits robust predictive capabilities across diverse operational scenarios. This enables the system to intelligently optimize energy management, enhance operational efficiency, and minimize energy losses, costs, and environmental emissions.

Furthermore, the approach supports dynamic modulation of energy demand in response to forecasted supply conditions, contributing to peak-load mitigation and reinforcing overall system stability. These results underscore the superior performance of the ANN-based AL strategy in resolving intricate techno-economic trade-offs. It consistently delivers well-balanced solutions characterized by heightened predictive accuracy, lower operational expenditures, reduced emissions, and improved system reliability.

**Fig. 13:**
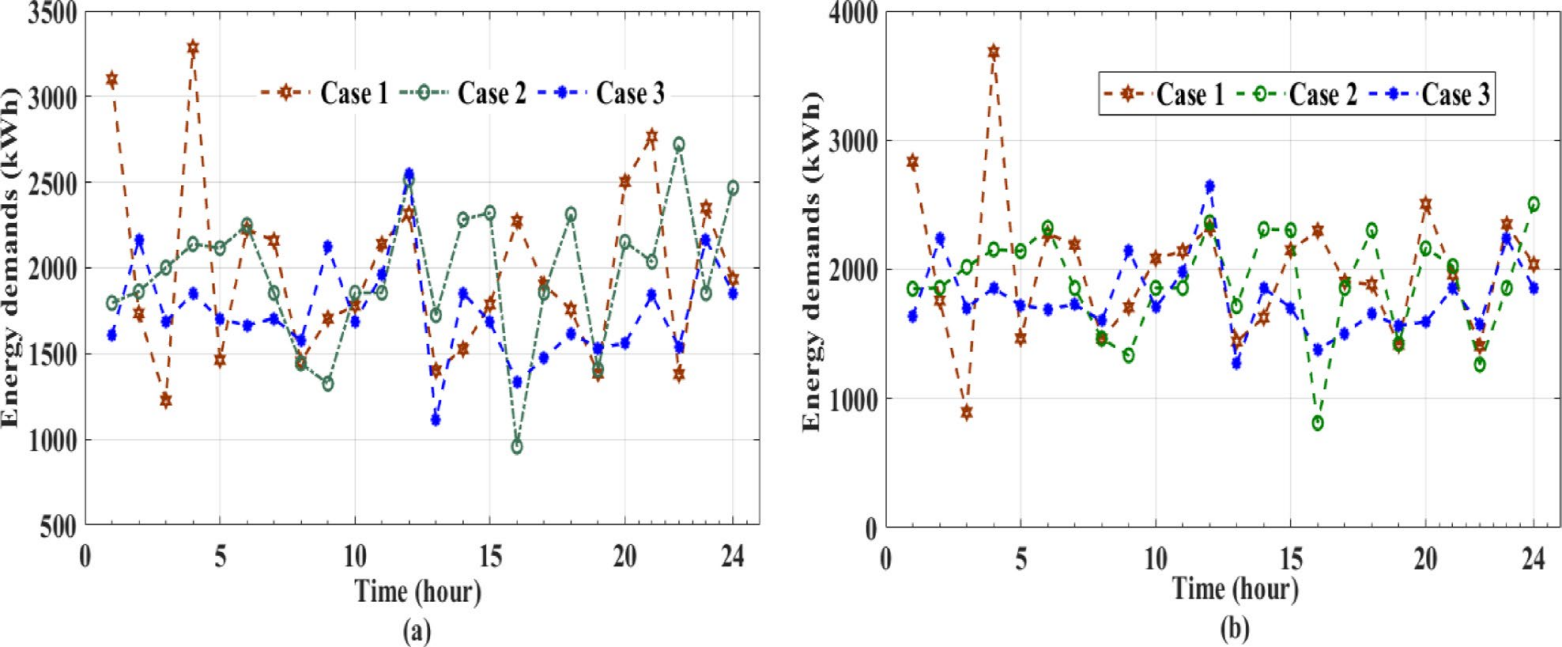
Performance for the three case studies for minimum deviation: **(a)** Optimal level for future energy demands, and **(b)** adjusted energy demand.

**Fig. 14:**
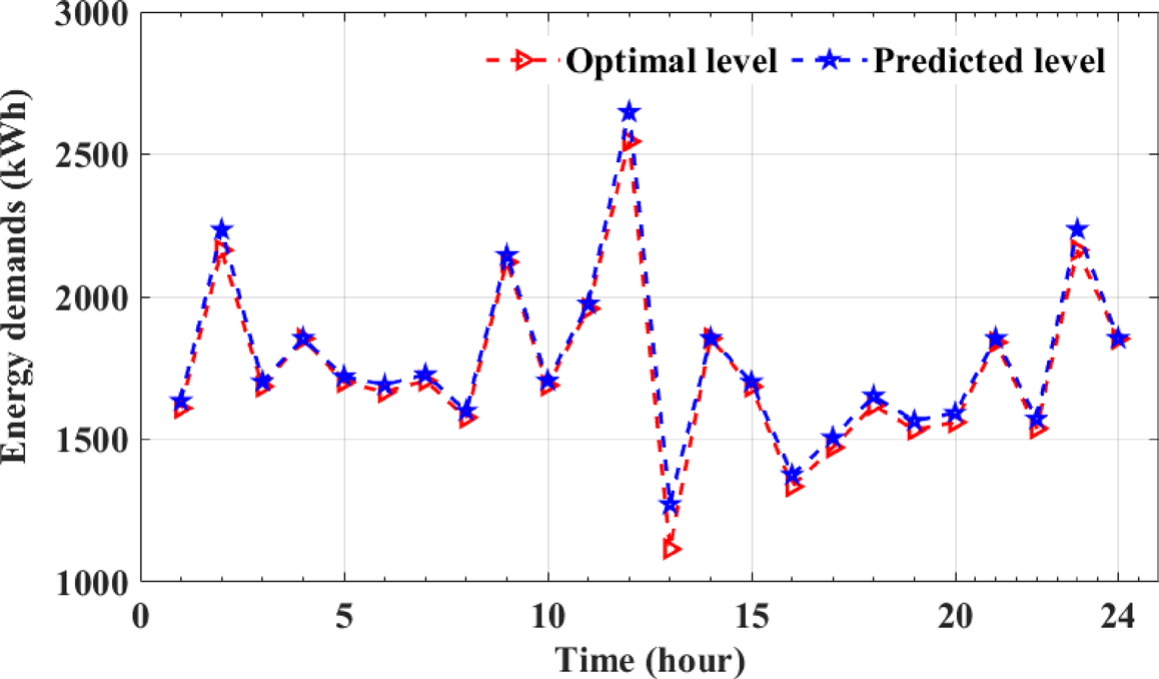
The deviation between future energy demand and optimal energy level using an ANN-based AL approach with uncertainty.

Figure 13 indicates that Case 1 provides more frequent fluctuations in energy demand, resulting in more deviations and less stability. Unlike in Cases 2 and 3, it produces fewer fluctuations in energy demand, especially in Case 3. Its performance has smoother, more consistent profiles, which ensures system stability and efficient energy management. The proposed approach’s effectiveness in accurately predicting future demand with uncertainty, effectively aligning with optimal energy levels, and maintaining minimal deviation ensures its reliability and capability to adapt to continuously changing energy demands, as illustrated in Fig. 14.

**Fig. 15:**
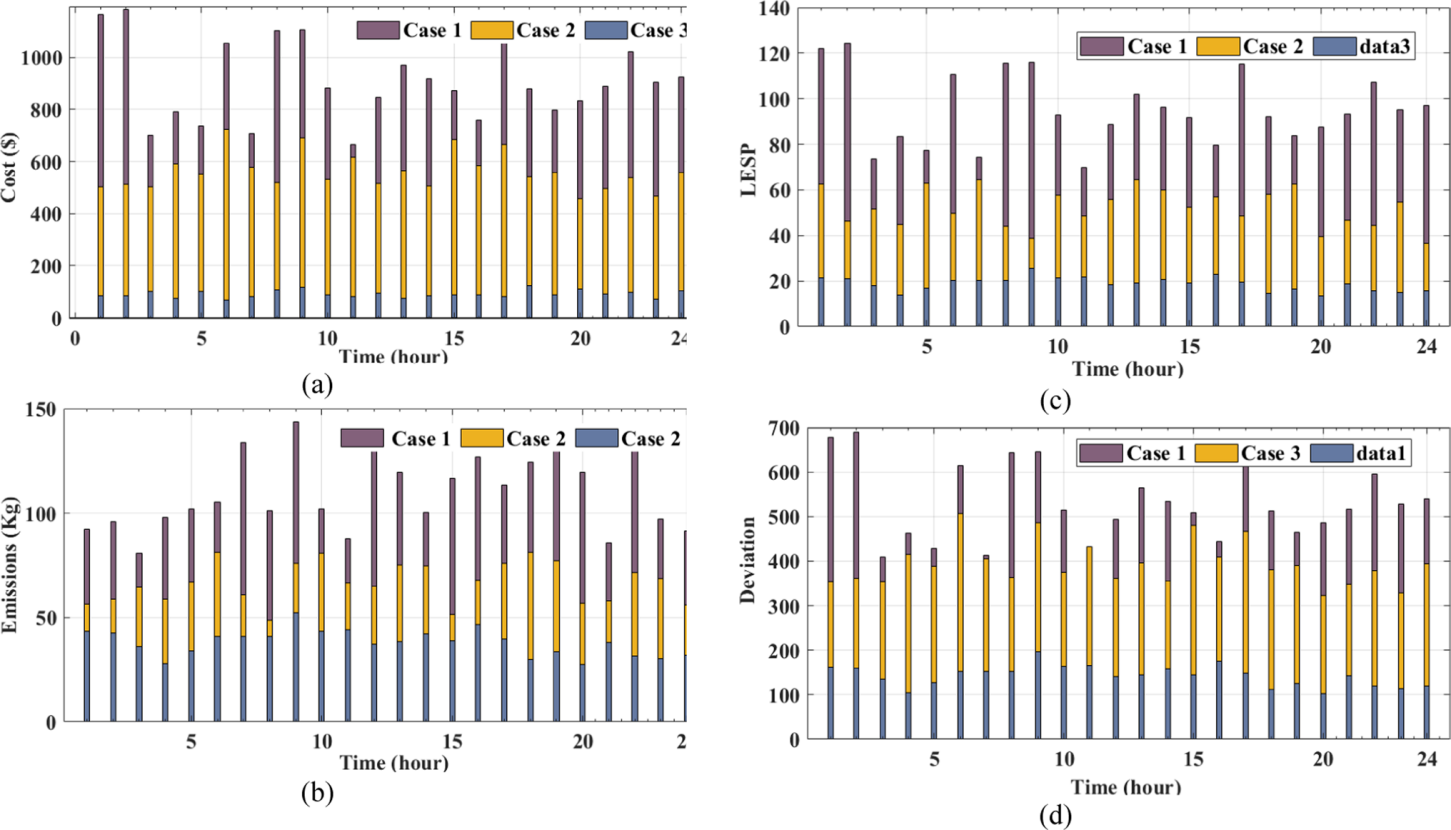
Muti-objective functions framework for EH across the three case studies: **(a)** cost minimization, **(b)** emission reductions, **(c)** LESP minimization, **(d)** deviation minimization.

The cost-effectiveness of the EH system is achieved through the reduction of operational expenditures and the maximization of self-consumption, following the identification of an optimal solution that addresses techno-economic trade-offs. This economic advantage is further reinforced by the system’s ability to adaptively manage resources while maintaining operational efficiency. Figure 15 presents a comprehensive analysis of the EH’s performance across the different case studies, evaluating their influence on operational costs, emissions, LESP, and deviations between actual energy demands and their optimal targets over a 24-hour horizon.

Achieving an optimal balance between power generation and load demand is essential for maximizing the efficient utilization of BS systems and EVs within their operational constraints. Enhancing energy efficiency is critical not only for maintaining system reliability and sustainability but also for addressing the inherent techno-economic trade-offs faced by modern energy systems. This study systematically evaluates the impact of uncertainty on the performance and reliability of EHs across three distinct scenarios.

In Case 1, the system demonstrates a high dependency on electricity procurement from the grid, leading to elevated operational costs, increased carbon emissions, substantial deviations between forecasted and actual energy demands, and a heightened risk of unplanned outages. Case 2 introduces a multi-objective optimization framework under uncertainty, yielding improved system performance through cost reductions and enhanced operational reliability. Case 3 advances this approach by incorporating an uncertainty-aware learning mechanism that iteratively refines the training dataset. This enables adaptive scheduling, optimized performance, and improved system resilience under fluctuating conditions.

The findings further emphasize the pivotal role of BS and EVs in mitigating the adverse effects of operational uncertainties. Their dynamic capabilities significantly contribute to maintaining system stability and elevating overall energy efficiency. As uncertainties in energy consumption patterns driven by increasingly variable consumer behavior achieve significant scheduling and management challenges for EHs, the study incorporates hourly demand fluctuations into the optimization framework. This integration fosters a robust and adaptive operational strategy that ensures consistent performance across variable conditions. Ultimately, the results validate the proposed methodology, demonstrating that Case 3 delivers superior outcomes across all key performance indicators. These include significant reductions in operational costs and emissions, improved supply reliability, and enhanced system efficiency, underscoring the model’s robustness and suitability for real-world EH applications under uncertainty.

It is indicated that in Fig. 15(a), the third case study records significant cost minimizations, which ensure economic efficiency while sustaining flexible operations. Figure 15(b) illustrates the reduction in emissions across three case studies and records the most significant minimizations with the proposed approach, which ensures their effectiveness in addressing environmental aspects. The first case study records the highest LESP values, while the third one consistently has the lowest LESP, as in Fig. 15(c). The performance of EH with ANN-based AL approach consistently has the lowest value of deviations, enhancing its superiority in power system optimization as in Fig. 15(d).

The performance of EH under the three case studies is shown in Table 4, which presents the techno-economic analysis for each case as follows:

### • Case 1

records that the performance of a single objective optimizing approach under an uncertainty framework provides moderate enhancements in performance overall by a 50.25% reduction in LESP value and achieving electrical and thermal deviations by 6.454 kW/day and 5.001 kW/day, respectively, with operating cost savings around 20,345.62 $/day and emissions minimization around 81,434.91 kg/day, in addition to the optimized output reaches 4765.9 kW/day.

### • Case 2

records that the performance of a multi-objective optimizing approach under uncertainty provides better improvements compared with Case 1, achieving reductions in LESP value by 76.04% and reducing electrical and thermal deviations to 4.684 kW/day and 2.947 kW/day, respectively, with operating cost savings around 39,946.33 $/day and reducing emissions by 56,580.30 kg/day, in addition to the enhanced optimized output reaching 8314.1793 kW/day.

### • Case 3

records that the performance of the proposed approach provides remarkable improvements that ensure system reliability and increase its efficiency by reducing LESP value to 94.7% and electrical and thermal deviations limited to 1.453 kW/day and 898.27 W/day, respectively, with operating cost savings increased to 62,734.21 $/day while minimizing emissions to 23,739.17 kg/day, in addition to providing the highest optimized output for 13,687.8 kW/day.

**Table 4:** Impact of the proposed techniques on the EH from an economic perspective.

Case study	1	2	3
**Initial LESP**	0.427960	0.427960	0.427960
**New LESP**	0.213506	0.102739	0.010682
**Improvement percentage (%)**	50.25	76.04	94.7
**Electrical deviation (kW/day)**	6.453790	4.6840.351771	1.453392
**Thermal deviation (kW/day)**	5.001447	2.946729577	0.898278
**Operation cost ($/day)**	108,289.3386	108,289.3386	108,289.3386
**Cost minimization ($/day)**	87,943.7144	68,343.0065	45,555.129
**Improvement percentage (%)**	18.8	36.9	57.9
**Total emissions (kg/day)**	120,423.1237	120,423.1237	120,423.1237
**Emissions reduction (kg/day)**	81,434.9119	56,580.297	23,739.1717
**Improvement percentage (%)**	32.4	53.01	80.3
**Optimized output (kW/day)**	4,765.9	8,314.1793	13,687.8

Finally, Case 3 provides the most efficient approach, providing an optimal balance between the techno-economic metrics. These findings highlight the effectiveness of an ANN-based AL approach to enhance the system’s reliability and provide optimal energy management with uncertainty and continuously changing energy demand.

## Conclusion

The combining of ANN-based AL with uncertainty into a multi-objective framework to address techno-economic trade-off challenges in EHs provides superior performance in enhancing the EH’s flexibility and sustainability during peak-demand periods and ensuring reliable operation. The AL helps ANN improve its predictive ability. It allows models to benefit from predictive and adaptive abilities to allocate optimal resources, allowing efficient utilization and sustainable energy management under complex dynamic conditions. The proposed approach predicts optimal GDF in the EH to improve decision-making under operating conditions and achieve balances in techno-economic trade-offs by regulating gas distribution among generating units. It prioritizes efficient energy utilization and minimizes operating costs and emissions to ensure system reliability. In addition, the BS unit and EVs are becoming increasingly significant in addressing operational uncertainties and ensuring system reliability against frequent fluctuations in energy demand. The main contribution of this research is addressing the critical techno-economic trade-off challenges for enhancing EH operation and ensuring its efficiency as follows:

- Obtaining minimal operating costs while preserving high reliability is an essential trade-off challenge. Enhancing the ANN model’s predictive ability by the AL algorithm facilitates obtaining optimal solutions that guarantee reliability metrics with uncertainty. The results verify that the proposed approach satisfies this target by providing a 57.9% decrease in operating costs and lowering the LESP value to 0.010682, which maintains system reliability and reduces interruption risks as much as possible.
- Ensuring energy efficiency is simultaneous with sustaining system flexibility by adapting to frequent changes in load dynamics, intermittent RES supply due to its nature, and peak demand periods. In addition, ANN-based AL significantly adapts to uncertainties and provides remarkable improvements in minimizing electrical and thermal deviations limited to 1.453 kW/day and 898.27 W/day, respectively. These improvements validate the approach’s effectiveness and achieve the balance of flexible operation with efficient energy management to ensure sustainability for EHs.
- Despite uncertainties and RES’s intermittent nature, the AL has robust predictive capabilities that allow the ANN’s performance to process data and learn from system performance to maximize RES utilization and make modifications to demand needs according to its predicted outputs to maintain system reliability and reduce interruption risks as much as possible. In addition to reducing energy losses, costs by 57.9, and emissions by 80.3% while enhancing efficient utilization for RESs and reliable operation for EH.
- The proposed approach improves dynamic adaptation in EH and balances RESs and system stability, ensuring sustainability, flexibility, and cost-effective power management while balancing reliability and efficiency.
- It is demonstrated that the optimized output is increased to 13,687.8 kW/day, which proves high prediction and efficient allocation of energy resources to meet high dynamic load changes while ensuring EH operational efficiency.

By addressing these challenges within a multi-objective framework under uncertainty, the proposed approach offers an advanced solution that simultaneously satisfies both technical and economic requirements for optimizing EH operations. This approach facilitates the development of sustainable and adaptable energy systems capable of meeting dynamic demand with cost-effective power management while maintaining a balance between reliability and efficiency. Future research will aim to address essential techno-economic trade-offs in EHs operations by achieving an optimal balance among cost efficiency, system reliability, and resilience under uncertainty and abnormal operating conditions, such as instances where energy demands surpass the output of RESs. Furthermore, the research will assess the overall performance of EHs in effectively managing these challenges.

## Data Availability

Due to their large size, the datasets generated during the current study are not publicly available but are available from the corresponding author upon reasonable request.
